# Interfacial-confined coordination to single-atom nanotherapeutics

**DOI:** 10.1038/s41467-021-27640-7

**Published:** 2022-01-10

**Authors:** Limei Qin, Jie Gan, Dechao Niu, Yueqiang Cao, Xuezhi Duan, Xing Qin, Hao Zhang, Zheng Jiang, Yongjun Jiang, Sheng Dai, Yongsheng Li, Jianlin Shi

**Affiliations:** 1 Lab of Low-Dimensional Materials Chemistry, Key Laboratory for Ultrafine Materials of Ministry of Education, Frontier Science Center of the Materials Biology and Dynamic Chemistry, Shanghai Engineering Research Center of Hierarchical Nanomaterials, School of Materials Science and Engineering, East China University of Science and Technology, 200237 Shanghai, China; 2 State Key Laboratory of Chemical Engineering and School of Chemical Engineering, East China University of Science and Technology, 200237 Shanghai, China; 3 Shanghai Synchrotron Radiation Facility, Zhangjiang Lab, Shanghai Advanced Research Institute, Chinese Academy of Sciences, 201210 Shanghai, China; 4 Key Laboratory for Advanced Materials and Feringa Nobel Prize Scientist Joint Research Center, Institute of Fine Chemicals, School of Chemistry & Molecular Engineering, East China University of Science and Technology, 200237 Shanghai, China; 5 Key Laboratory for Green Processing of Chemical Engineering of Xinjiang Bingtuan, School of Chemistry and Chemical Engineering, Shihezi University, 832003 Shihezi, China; 6 State Laboratory of High Performance Ceramics and Superfine Microstructure, Shanghai Institute of Ceramics, Chinese Academy of Sciences, 200050 Shanghai, China

**Keywords:** Biomedical materials, Nanotechnology in cancer, Catalysis

## Abstract

Pursuing and developing effective methodologies to construct highly active catalytic sites to maximize the atomic and energy efficiency by material engineering are attractive. Relative to the tremendous researches of carbon-based single atom systems, the construction of bio-applicable single atom materials is still in its infancy. Herein, we propose a facile and general interfacial-confined coordination strategy to construct high-quality single-atom nanotherapeutic agent with Fe single atoms being anchored on defective carbon dots confined in a biocompatible mesoporous silica nanoreactor. Furthermore, the efficient energy conversion capability of silica-based Fe single atoms system has been demonstrated on the basis of the exogenous physical photo irradiation and endogenous biochemical reactive oxygen species stimulus in the confined mesoporous network. More importantly, the highest photothermal conversion efficiency with the mechanism of increased electron density and narrow bandgap of this single atom structure in defective carbon was proposed by the theoretical DFT calculations. The present methodology provides a scientific paradigm to design and develop versatile single atom nanotherapeutics with adjustable metal components and tune the corresponding reactions for safe and efficient tumor therapeutic strategy.

## Abstract (web summary)

Developing single atom systems with improved catalytic potential for bio-application has major therapeutic potential. Here, the authors report on the development of a metal single-atom on a carbon dot support confined within mesoporous silica for the development of therapeutic agents.

## Introduction

The high efficiency and economy of matter utilization and energy conversion in living organisms is astonishing. The metabolic pathways such as glycolysis and the citric acid cycle, occur through step-by-step chemical reactions catalyzed by single metal active center in enzyme or coenzyme, to produce their end products efficiently and in a minimal number of steps^1–3^. The efficient use of active metal centers and the maximum energy conversion has become the optimal simulation methodology in the development of precise synthesis and application. Single-atom catalysis is a powerful and attractive technique with exceptional performance, drastic cost reduction and notable catalytic activity and selectivity^4–7^. In single-atom catalysis, supported single-atom catalysts contain isolated individual atoms on certain supports, showing the extraordinary activity, good stability, and excellent atomic economy due to the highly exposed, dispersed catalytic active centers^8^.

Various advanced strategies such as impregnation and coprecipitation, spatial confinement and coordination site construction have been developed to synthesize carbon-based metal single atom systems^9^. Especially, the nitrogen-coordinated metal single atoms on carbon-supported catalysts (M-N-C) are the most promising single atom materials, whose performances always depend on the synthesis approaches such as the mixing procedures among carbon, nitrogen and metal precursors, and the annealing step to obtain M-N_x_ active moiety doped in porous carbon framework^10–12^. These single atom systems exhibited maximum atom catalytic efficiency and low cost, which have been tremendously investigated in oxygen reduction reaction (ORR)-related catalytic fields with promising application potentials^13–18^. Relatively, few reports of metal single atom-anchored nanoplatforms have been constructed, specifically carbon-dot-supported dispersed gold particles, iron or zinc single atom-dispersed carbon spheres and copper single atom-doped hollow carbon sphere were elaborately engineered to catalyze the conversions of substrates into the reactive oxygen species in tumor environment^19–22^. Besides, the relationship between the precise synthesis of M-N-C structure and its biological effects in nanomedicine is still unclear and need to be further explored, which can influence the mass transport property and thereafter the utilization of the active sites. Thus, developing effective methodologies for the construction of high-quality metal single-atom nanotherapeutic agents with both good biocompatibility and high bio-stability to maximize the atomic and energy efficiency is still a great challenge.

**Fig. 1:**
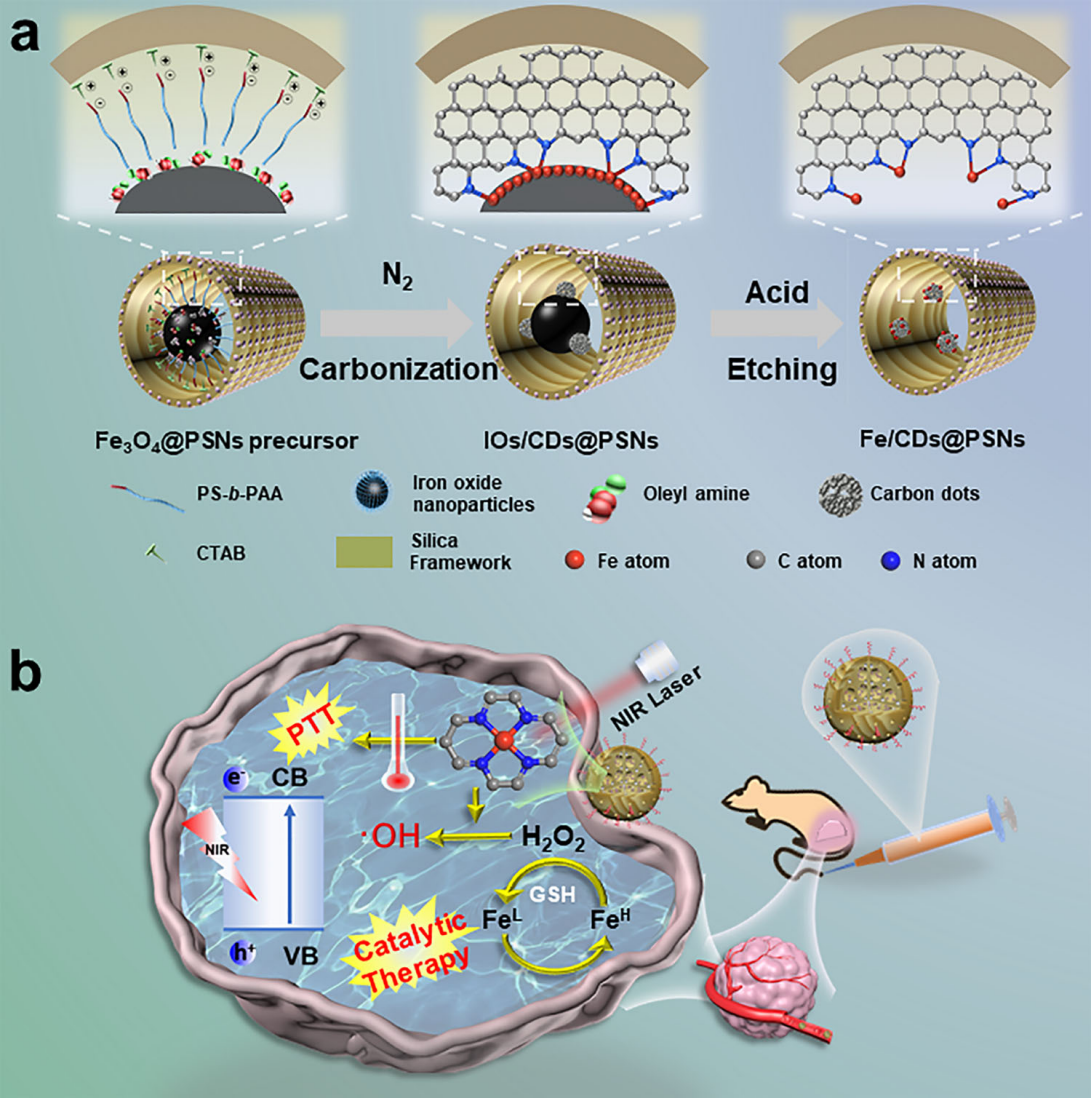
Schematic illustration for the synthesis and biological effect of Fe/CDs@PPSNs. **a** Synthetic procedure. **b** Therapeutic effects by combined photothermal and catalytic tumor therapy.

Herein, we develop a simple but efficient interfacial-confined coordination strategy to construct Fe single atoms-anchored defective carbon dots in PEGylated porous silica nanoreactors (designated as Fe/CDs@PPSNs). The proposed strategy is based on the in-situ high-temperature carbonization of polymers/N-containing molecules (i.e. polystyrene-*b*-poly(acrylic acid) (PS-*b*-PAA), cetyltrimethylammonium bromide (CTAB) and oleylamine) to nitrogen-doped carbon dots and the interfacial coordination between N and Fe atoms confined at the interface between the carbon dots and iron oxide nanoparticles, followed by acid etching to remove the redundant iron-based nanoparticles in a biocompatible porous nanoreactor (Fig. 1a). Furthermore, the efficient energy conversion capability of the silica-based Fe single atoms systems in terms of photothermal and Fenton catalytic tumor therapy has been demonstrated under the exogenous physical photo irradiation and endogenous biochemical reactive oxygen species (ROS) stimulus. The confined coordination approach has three significant features: the chemical coordination takes place within the mesopore channels, where the Fe-N-C single atoms are formed by the strong coordination effects between uniform-distributed iron oxide nanoparticles and nitrogen-doped defective carbon dots originated directly from the confined carbonization of organic templates/components in the uniform and connected hierarchical mesopores, following by acid etching to remove redundant iron oxide nanoparticles, resulting in the successful formation of Fe-N-C single atoms in biocompatible porous silica nanoreactors. The biological reactions are confined in the mesopore channels (Fig. 1b): the confined Fe-N-C of increased carbon electron density and reduced energy level led to the efficient photothermal conversion under 808 nm laser irradiation. Besides, in the interconnected porous framework, the dual-activated pH/GSH Fenton reaction of Fe-N-C moieties can be realized with highly efficient substrate transport, to catalyze tumor over-expressed H_2_O_2_ to produce hydroxyl free radicals for safe and effective tumor-specific therapy. The composition adjustability in the mesopore channels: the tailorable hydrophobic interaction induced self-assembly between PS blocks and oil-soluble functional nanocrystals makes the composition adjustable. A class of nitrogen-coordinated metal single-atom nanotherapeutic agents (M-N-C) can be obtained by encapsulating varied hydrophobic metal-based cores in the biocompatible silica mesoporous channels via this general confined coordination approach.

## Results and discussion

### Synthesis and characterizations of Fe/CDs@PPSNs

**Fig. 2:**
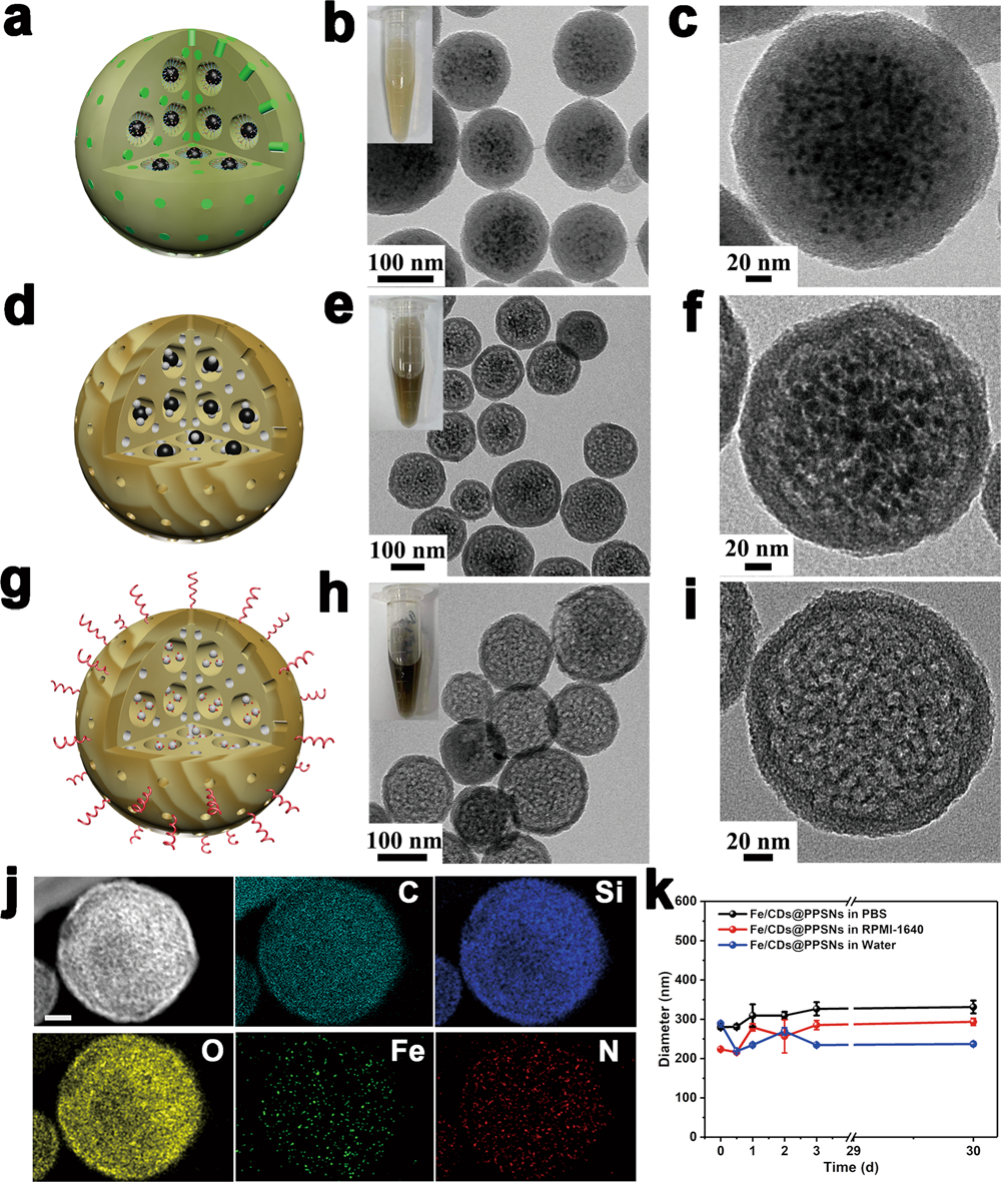
Characterizations of Fe/CDs@PPSNs. Schematic diagram, TEM images and digital photographs (up-left insert) of (**a**–**c**) Fe_3_O_4_@PSNs precursor, (**d**–**f**) IOs/CDs@PSNs, and (**g**–**i**) Fe/CDs@PPSNs. **j** STEM-EDS element mappings (C, Si, O, Fe, N) of Fe/CDs@PPSNs (Scale bar = 50 nm). **k** Stability of Fe/CDs@PPSNs in RPMI-1640, PBS and water for 30 days. The data are expressed as means ± s. d. from three independent replicates.

The well-crystallized and spherical hydrophobic magnetite (Fe_3_O_4_) nanoparticles were prepared via thermal decomposition method (Supplementary Figs. 1 and 2)^23^ .Then, Fe_3_O_4_@PSNs precursor with an average particle size of ~200 nm was synthesized through the co-assembly between Fe_3_O_4_, PS-*b*-PAA, and CTAB and the subsequent hydrolysis and condensation of TEOS under basic conditions (Fig. 2a). The black Fe_3_O_4_ dots were uniformly distributed, verifying the successful confinement of organic template-capped hydrophobic Fe_3_O_4_ nanoparticles in the PS-*b*-PAA/CTAB-filled large pore channels through the hydrophobic interaction (Fig. 2b, c). After carbonization in the nitrogen flow, the resultant iron oxide nanoparticles and carbon dots co-encapsulated porous silica nanoparticles (designated as IOs/CDs@PSNs) maintained its spherical morphology and good monodispersity (Fig. 2d–f). Notably, the aqueous solution of IOs/CDs@PSNs exhibits dark brown color (up-left inset of Fig. 2e), mainly derived from the extensive carbonization of organic templates (PS-*b*-PAA and CTAB) in the large pores. After acid etching and subsequent PEG modification, the Fe/CDs@PPSNs were finally obtained (Fig. 2g–i).

The successful removal of iron oxide by acid etching was confirmed by TEM and XRD patterns (Fig. 2h and Supplementary Fig. 3). In addition, PEG grafting was characterized by FT-IR spectra and zeta potential (Supplementary Fig. 4). STEM-EDS element mappings of Fe/CDs@PPSNs reveal that Si, O, Fe, C, and N are evenly dispersed in the whole structure (Fig. 2j) with the C and N amounts of 6.16 wt% and 0.75 wt% determined by quantitative element analyses, respectively. The loading amount of Fe element was quantified to be 0.3 wt% by inductively coupled plasma optical emission spectrometry (ICP-OES). N_2_ adsorption-deposition isotherms (Supplementary Figs. 5 and 6) of Fe/CDs@PPSNs and IOs/CDs@PSNs exhibit a typical type-IV hysteresis, indicating the presence of uniform dual-mesoporous structure (1.8 nm and 13.7 nm). The BET surface area and pore volume of Fe/CDs@PPSNs were calculated to be 518 m^2^/g and 0.95 cm^3^/g, respectively (Supplementary Table 1), which are significantly higher than those of IOs/CDs@PSNs due to the existence of iron oxide nanoparticles in the pore channels. As shown in Fig. 2k, Fe/CDs@PPSNs exhibit excellent long-term stability in PBS and cellular culture medium even after one month. Actually, the mesoporous network structure plays a vital role in improving the dispersion of Fe and carbon species, as evidenced by the fact that the control samples of IOs/CDs and Fe/CDs without the silica porous network show the larger hydrodynamic sizes with severe particle agglomeration in the aqueous environment (Supplementary Fig. 7).

### Characterizations of Fe state in Fe/CDs@PPSNs

**Fig. 3:**
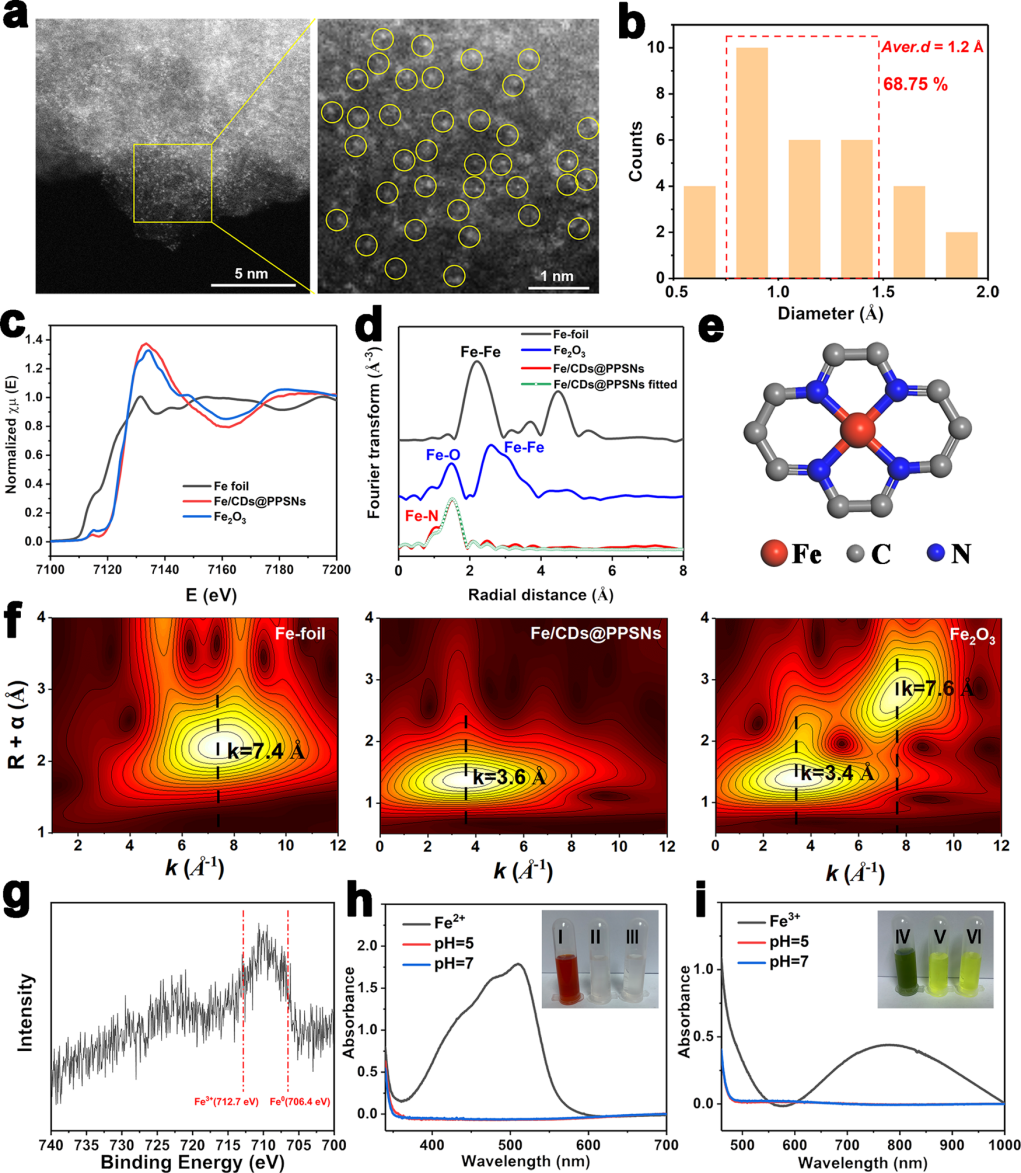
Characterizations of Fe state in Fe/CDs@PPSNs. **a** Aberration-corrected HADDF-STEM images of Fe/CDs@PPSNs after the etching by NaOH. The bright dots indicate the highly dispersive Fe single atoms in the carbon matrix. **b** Histogram diameter distribution of iron species. Data were collected using Digital Micrograph software. **c** Fe K-edge XANES spectra of Fe foil, Fe_2_O_3_, and Fe/CDs@PPSNs. **d** Fourier transformation EXAFS spectra of Fe foil, Fe_2_O_3_, and Fe/CDs@PPSNs in R-space. **e** The proposed Fe-N_4_-C local environment. **f** Wavelet transformation EXAFS spectra of Fe foil, Fe_2_O_3_, and Fe/CDs@PPSNs. **g** Fe2*p* XPS spectrum of Fe/CDs@PPSNs. Detection of ferrous and ferric ions by o-phenanthroline **h** and K_3_Fe (CN)_6_ **i** agents at different pH values. Insets in **h** and **i**: (I-III) Digital photos of ferrous ions with o-phenanthroline and treating Fe/CDs@PPSNs with o-phenanthroline at pH=5 and pH=7, respectively; (IV-VI) Digital photos of ferric ions with K_3_Fe (CN)_6_ and treating Fe/CDs@PPSNs with K_3_Fe (CN)_6_ at pH=5 and at pH=7, respectively.

The Fe single atoms in Fe/CDs@PPSNs have been further determined by the aberration-corrected HADDF-STEM measurement. After NaOH etching, the high-density of Fe single atoms marked with yellow circles can be clearly identified on the carbon support (Fig. 3a). The average diameter of these bright dots was measured to be 1.2 Å (Fig. 3b), which is substantially smaller than the length of iron-iron covalent bond (*d*_Fe_ = 2.74 Å), validating the atomically dispersed Fe atoms on the support^14^. X-ray absorption spectra (Fig. 3c) show the first inflection point E_0_ located at the position (i.e., 7131.5 eV) between those of Fe foil and Fe_2_O_3_, demonstrating that the valence of Fe single atoms in Fe/CDs@PPSNs is higher than of the metallic Fe but lower than that of Fe^3+^ (named as Fe^δ+^, 0<δ < 3)^24^. Fourier transformed extended X-ray absorption fine structure (FT-EXAFS) spectrum in Fig. 3d shows the main peak at ~1.97 Å, which can be assigned to the scattering interaction between Fe and N/O in the first-shell Fe-N(O). In addition, the peak associated with the scattering interaction between Fe atoms and Fe the first shell (Fe-Fe) is not observed, suggesting the presence of Fe single atoms^25^. Fe atoms are coordinated with 4.4 ± 0.2 N atoms from the EXAFS fitting results (Supplementary Table 2), revealing the coordination structure of Fe-N_4_ in Fe/CDs@PPSNs (Fig. 3e). As demonstrated by the wavelet transformed (WT) contour plots of Fe foil and Fe_2_O_3_ (Fig. 3f), the intensity maxima at ~7.4 and ~3.4 Å are assigned to the contributions of Fe-Fe and Fe-O bonds, respectively. In contrast, the WT contour plot of Fe/CDs@PPSNs displays only one intensity maximum at around ~3.6 Å, which is attributed to the Fe-N (O) contribution^26^. These results demonstrate the presence of Fe single atoms and the absence of clusters and nanoparticles in Fe/CDs@PPSNs.

To confirm the chemical state of Fe and the chemical environments of N in the Fe/CDs@PPSNs, X-ray photoelectron spectroscopy (XPS) analysis was performed and four elements of C, N, Fe, O are found (Supplementary Fig. 8 and Table 3). In detail, an obvious peak between Fe^0^ (706.4 eV) and Fe^3+^ (712.7 eV) is observed in the Fe *2p* XPS spectrum (Fig. 3g), proving that the valence state of iron in Fe/CDs@PPSNs is between 0 and 3, in accordance with the above XANES analysis. In addition, the N*1s* spectrum of Fe/CDs@PPSNs is deconvoluted into three peaks centered at 398, 399.3, and 402.7 eV (Supplementary Fig. 9), which are assigned to pyridinic N (or Fe-N), pyrrolic N, and oxidized N^27^, respectively. Among these, pyridinic N specie can serve as effective anchor sites for the stabilization of Fe single atoms in the Fe/CDs@PPSNs^28^. It is noted that the intensities of both Fe and N peaks is relative weak, which is probably owing to the low iron and N contents in Fe/CDs@PPSNs. Besides, in comparison to the obvious leakage of iron species from the control sample of IOs/CDs@PPSNs (Supplementary Fig. 10), Fe single atoms in the Fe/CDs@PPSNs exhibit high stability, and no leaching of ferrous or ferric ions can be detected even incubated in a mild acidic environment for 3 days (Fig. 3h, i) due to the strong binding ability of Fe-N bonds in the pore channels.

It is known that the transition metals (M = Mn, Fe, Ni, etc.) possessing the 3*d* unoccupied orbitals can accommodate foreign electrons to form the stable metal-nitrogen complex carbon (M-N-C) materials. By manipulating the assembly behaviors of various metal sources and surfactants, a series of metal single atoms in the confined pore channels are expected by the proposed interfacial-confined coordination strategy. For this purpose, both Ni/CDs@PPSNs and Mn/CDs@PPSNs with Ni and Mn single atoms in the porous silica nanoreactor have been successfully prepared, as verified by TEM, STEM-EDS element mapping and aberration-corrected HADDF-STEM images (Supplementary Figs. 11, 12 and 13). To verify the universality of the proposed strategy, density functional theory (DFT) calculations show that the values of binding energy (Eads) for the Fe-N_4_-C, Ni-N_4_-C and Mn-N_4_-C moieties are −2.23 eV, −4.98 eV, and −1.44 eV, respectively (Supplementary Figs. 14 and 15). This indicates that the N_4_-C moiety in the present work is thermodynamically favorable for anchoring other metal single-atoms. Consequently, this interfacial-confined coordination strategy provides a unique and general pathway to convert metal oxide nanoparticles to metal single-atoms in the confined biocompatible silica porous channels for biomedical applications.

### Photothermal performance of Fe single atom-anchored defective carbon in Fe/CDs@PPSNs and DFT calculations

**Fig. 4:**
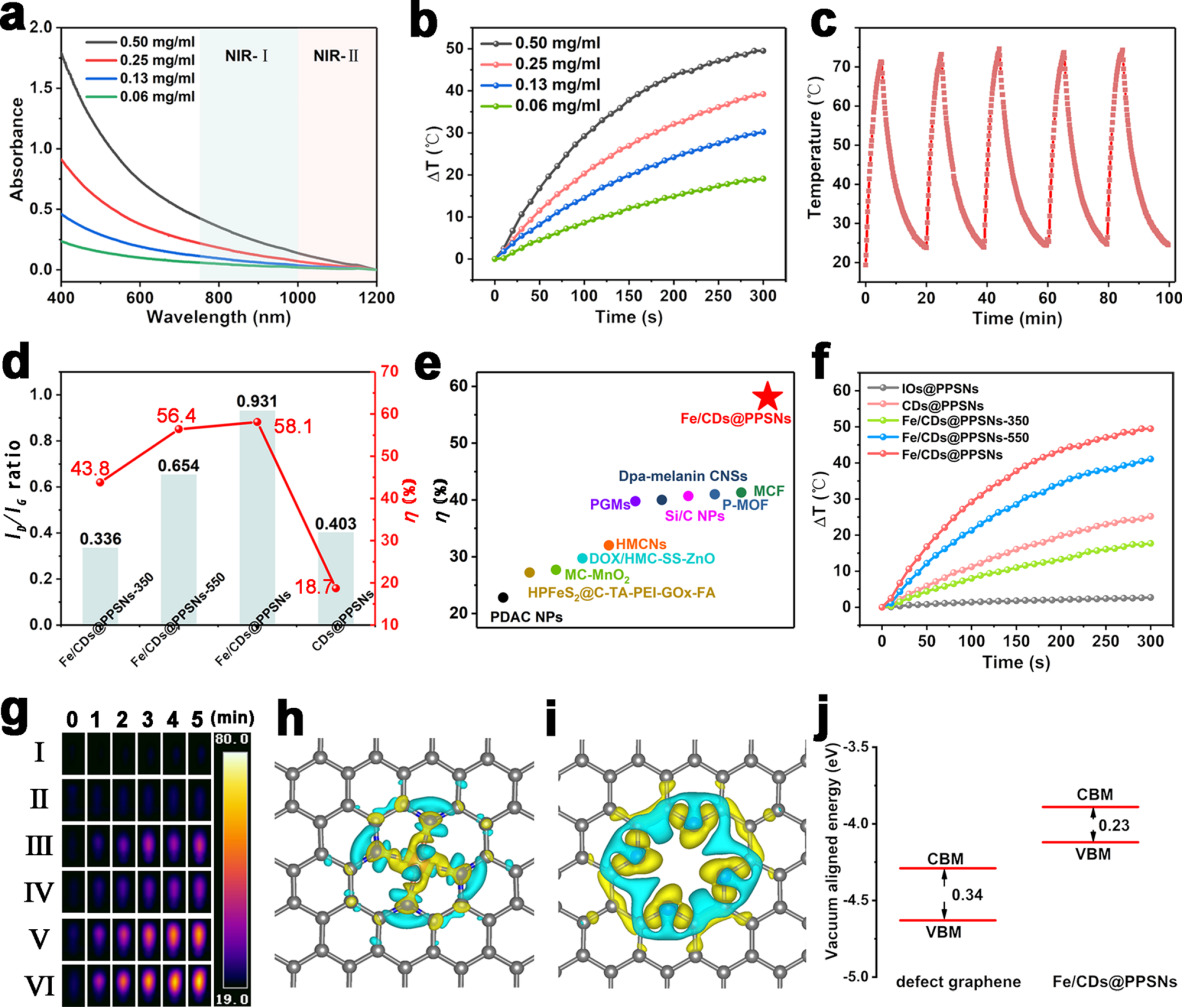
Photothermal performance and DFT calculations. **a** UV-vis-NIR spectra of Fe/CDs@PPSNs at different concentrations. **b** Photothermal heating curves for Fe/CDs@PPSNs dispersions at different dispersion concentrations (808 nm, 2 W/cm^2^). **c** Heating curves of Fe/CDs@PPSNs dispersion (0.5 mg/ml) in water for five laser on/off cycles under a NIR laser (808 nm, 2 W/cm^2^). **d** *I*_D_/*I*_G_ ratios (derived from the Raman spectra) and photothermal conversion efficiencies of Fe/CDs@PPSNs-350, Fe/CDs@PPSNs-550 Fe/CDs@PPSNs and CDs@PPSNs. **e** Temperature increment and photothermal conversion efficiencies of various carbon-based PTT agents. Photothermal heating curves (**f**) and IR images (**g**) of I: H_2_O (control) and an aqueous dispersion of II: IOs@PPSNs, III: CDs@PPSNs, IV: Fe/CDs@PPSNs-350, V: Fe/CDs@PPSNs-550, and VI: Fe/CDs@PPSNs during NIR laser irradiation for 5 min (0.5 mg/ml, 808 nm, 2 W/cm^2^). The charge density difference between (**h**) Fe/CDs@PPSNs and (**i**) defect graphene. Yellow and light blue isosurfaces denote an increment by 0.005 e/Å^3^ and a reduction by 0.005 e/Å^3^ of electronic density, respectively. (**j**) Energy levels of the frontier orbitals for defect graphene and Fe/CDs@PPSNs.

The aqueous solution of Fe/CDs@PPSNs exhibits board absorption ranging from UV to NIR region (Fig. 4a). The temperature can be elevated by about 49.5 °C under 808 nm laser irradiation at a power density of 2.0 W/cm^2^ for 5 min of Fe/CDs@PPSNs at 0.50 mg/mL (Fig. 4b), while the temperature of pure water was increased by only 1.6 °C (Supplementary Fig. 16). The photothermal heating effect of Fe/CDs@PPSNs shows excellent photostability (Fig. 4c) and a laser power-dependent manner (Supplementary Fig. 17). Following the reported calculation method by Roper et al.^29^, the optimal photothermal conversion efficiency of Fe/CDs@PPSNs can be calculated to as high as 58.1% (Fig. 4d), which is much higher than that of most amorphous carbon-based and other kinds of photothermal generators (Fig. 4e and Supplementary Tables 4 and 5). The intensity ratio of D-band at 1340 cm^−1^ to that of G-band at 1570 cm^−1^ (*I*_D_/*I*_G_) from the Raman spectrum of Fe/CDs@PPSNs, which represents the degree of carbon defects^30,31^, was calculated to be 0.931 (Fig. 4d and Supplementary Table 6). More importantly, the temperature of Fe/CDs@PPSNs was increased by 48 °C under 1064 nm exposure (Supplementary Fig. 18), verifying the great potentials for further efficient deeper tissue photothermal therapy.

To further clarify the photothermal contribution of Fe/CDs@PPSNs, the control samples of carbon-free IOs@PPSNs and iron-free CDs@PPSNs were prepared (Supplementary Fig. 19), with the similar particle sizes and pore structures to those of Fe/CDs@PPSNs (Supplementary Figs. 20–22). No significant temperature increment was observed for the IOs@PPSNs (Fig. 4f, g), indicating the main contribution of photothermal effect from carbon species. The element analysis results show that the carbon contents of CDs@PPSNs and Fe/CDs@PPSNs with remarkably stronger absorption intensity (Supplementary Fig. 23) are measured to 1.40 wt% and 6.16 wt%, respectively. CDs@PPSNs show about five times lesser carbon contents than that of Fe/CDs@PPSNs, indicating that Fe species can stabilize the carbon species and prevent the carbon loss during high-temperature pyrolysis. With the help of Fe coordination effects, more topological carbon defects generated in Fe/CDs@PPSNs and induced higher temperature increment at the same carbon concentration (Supplementary Fig. 24 and Table 7), suggesting that the Fe single-atom doping in carbon framework plays a critical role in improving the photothermal capability. In addition, the effect of carbonization temperature (350, 550, and 800 °C) on the heating performance of IOs/CDs@PSNs and Fe/CDs@PPSNs was intensively explored (Supplementary Figs. 25–28). Along with the increase of carbonization temperature, XPS peaks of Fe *2p* shifted to a higher binding energy level, implying the electron-deficient state of iron and electron-rich state of carbon. As expected, the Fe/CDs@PPSNs-350 and Fe/CDs@PPSNs-550 present the relatively poor photothermal conversion efficiencies and low *I*_D_/*I*_G_ ratios of Fe/CDs@PPSNs-350 and Fe/CDs@PPSNs-550 (Figs. 4d, f, g and Supplementary Figs. 29–31). These results demonstrate that the carbon defects of Fe/CDs@PPSNs can be easily tuned to improve the photothermal capability by changing the carbonization temperature.

The intrinsic photothermal mechanism of Fe/CDs@PPSNs was further analyzed by density DFT calculations of Bader charge and electronic band structure. The calculated charge density distributions show distinguishable charge transfer between Fe single atoms and defected graphene compared to the pristine defected graphene (Fig. 4h, i). For the pristine defected graphene, around 0.136 e is transferred from C atoms to N atoms, yielding electron-deficient C and electron-rich N atoms. In contrast, as high as 1.081 e is transferred from the Fe atom to the defected graphene in Fe/CDs@PPSNs, in which N atoms gain 0.186 e, similarly to that in the pristine defected graphene, in contrast to 0.895 e received by the C atoms. These verify that the N atoms can act as transfer medium of electron between Fe atom and C atoms, which gives rise to strongly electron-rich C atoms in Fe/CDs@PPSNs. The partial density of states (PDOS) on the Fe-N_4_-C and N_4_-C are shown in Supplementary Figs. 32 and 33. Results show that the band energy of the N atom at ca. −2 eV and 0 eV in Fe-N_4_-C obviously were weakened compared with the N atom in N_4_-C, and some more sharp peaks of Fe atom in the range of the band energy appeared. Moreover, there are interaction ranges between Fe and N atoms at ca. 2.2 eV and 3.2 eV. These suggest that the obvious orbital hybridization in the Fe 3d orbital and the N 1 s orbital in the Fe-N_4_-C, which is thus confirmative of the incorporation of Fe atoms in the N_4_-C moiety. Furthermore, the calculated band gap energies of Fe/CDs@PPSNs and CDs@PPSNs are 0.23 eV and 0.34 eV, respectively (Fig. 4j and Supplementary Figs. 34 and 35). Accordingly, the photons of 1.535 eV from 808 nm laser can be easily absorbed by Fe/CDs@PPSNs than CDs@PPSNs^32^, which leads to the fact that the electrons located in the VBM level can be readily excited into CBM level. Subsequently, the created excitons can recombine with the vacancy at VBM via a nonradiative process and release heat, which means that more electrons undergo electron-phonon coupling relax and more light energy is transferred to induce the vibration of the entire carbon lattice, leading ultimately to an increase of light-to-heat conversion efficiency^33^.Thus, the Fe single atoms-induced electron-rich carbon defects with the narrower bandgap in Fe/CDs@PPSNs are expected to promote the photothermal performance.

### Dual pH/GSH-triggered selectively catalytic activities of Fe/CDs@PPSNs

**Fig. 5:**
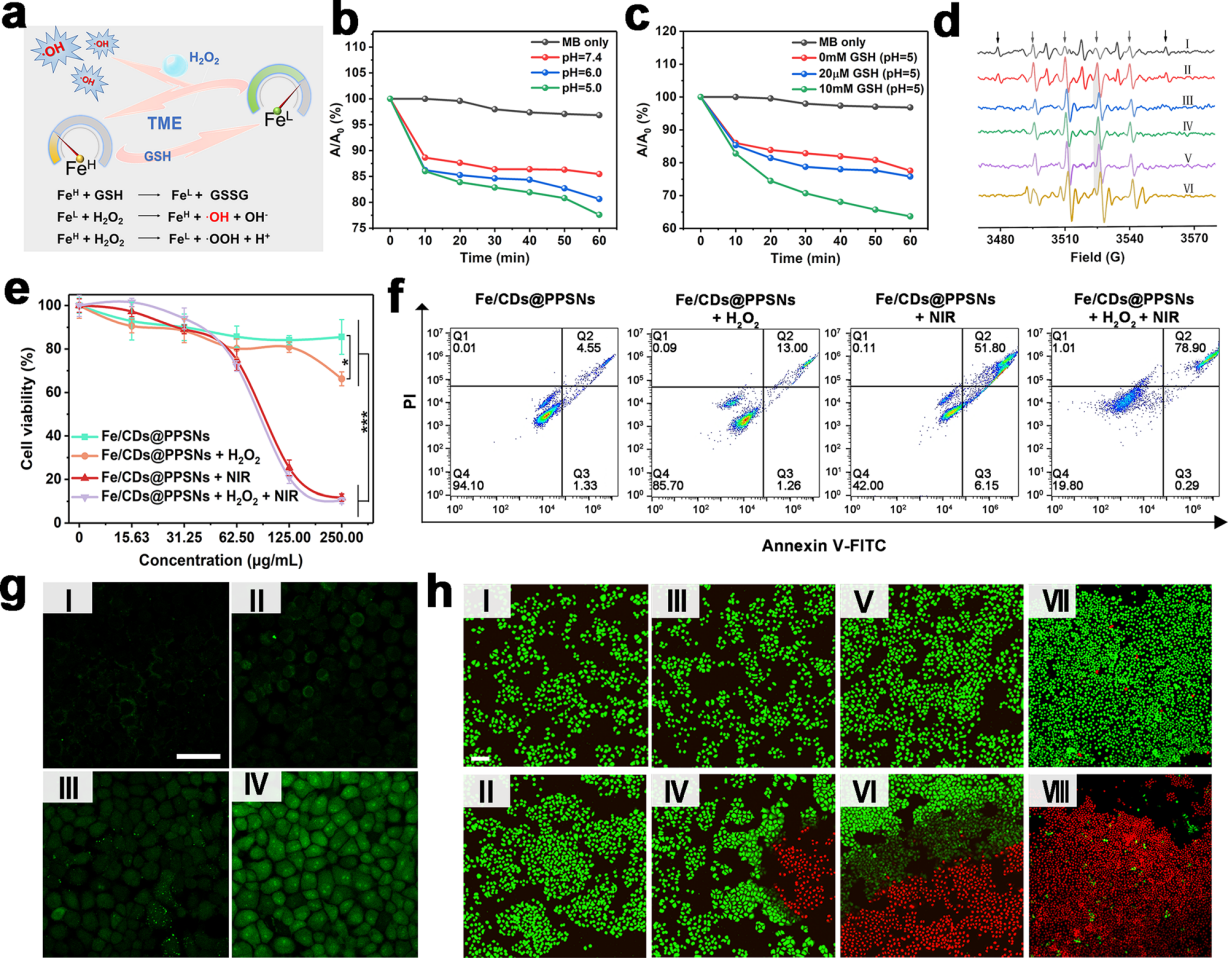
Dual pH/GSH-triggered selectively catalytic activities of Fe/CDs@PPSNs and the therapeutic effect of Fe/CDs@PPSNs against tumor cells. **a** Schematic diagram of tumor catalytic therapy of Fe/CDs@PPSNs. **b-c** MB degradation in PBS (10 mM) at various pH values (**b**) and GSH concentrations (**c**), respectively. **d** 5, 5-dimethyl-1-pyrroline N-oxide (DMPO) spin-trapping ESR spectra of Fe/CDs@PPSNs under different conditions (І: Fe/CDs@PPSNs (pH: 7.4), II: Fe/CDs@PPSNs + 100 µM H_2_O_2_ (pH: 7.4), III: Fe/CDs@PPSNs + 100 µM H_2_O_2_ (pH: 6.0), IV: Fe/CDs@PPSNs + 100 µM H_2_O_2_ (pH: 5.0), V: Fe/CDs@PPSNs + 100 µM H_2_O_2_ + 20 µM GSH (pH: 5.0), VI: Fe/CDs@PPSNs + 100 µM H_2_O_2_ + 10 mM GSH (pH: 5.0). **e** Cell viability by MTT assay and **f** apoptotic results by flow cytometry analyses of SMMC-7721 cells treated with Fe/CDs@PPSNs. **g** Detection of oxidative stress in SMMC-7721 cells after different treatments: (I) control; (II) H_2_O_2_; (III) Fe/CDs@PPSNs; (IV) Fe/CDs@PPSNs+H_2_O_2_ (scale bar: 50 µm). **h** Staining of living/dead SMMC-7721 cells by different treatments: (I) control group, (II) laser irradiation only, (III) CDs@PPSNs, (IV) CDs@PPSNs + NIR, (V) Fe/CDs@PPSNs and (VI) Fe/CDs@PPSNs + NIR, (VII) Fe/CDs@PPSNs + 100 µM H_2_O_2_, (VIII) Fe/CDs@PPSNs+100 µM H_2_O_2_ + NIR, all groups were stained with calcein AM and PI, where green fluorescence from calcein AM and red fluorescence from PI indicate living and dead cells, respectively (scale bar: 50 µm). The data in **e** are expressed as means ± s. d. from three independent replicates. *p* values were analyzed by Student’s two-sided *t*-test (*p < 0.05, **p < 0.01, ***p < 0.001).

As shown in Fig. 5a, a dual pH/GSH-triggered TME-responsive catalytic pathway of Fe/CDs@PPSNs is proposed. It was reported that the enhanced affinity of H_2_O_2_ to Fe sites at low pH and the increase of reactive low-valence Fe from high-valence Fe by the endogenous GSH can significantly contribute to the high Fenton activity of Fe-based nanoplatform^19,34^. To verify this hypothesis, as shown in Fig. 5b, c, the remarkable decreases of methylene blue (MB) absorbance both in acid conditions (pH=6.0 and 5.0) and after the addition of GSH indicate the •OH generation by Fe/CDs@PPSNs under the presence of H_2_O_2_. The strongest ·OH signals (1:2:2:1 multiple peaks) are observed in the electron spin resonance (ESR) spectra of Fe/CDs@PPSNs added with 10 mM GSH (pH=5.0) and 100 μM H_2_O_2_ (Fig. 5d)^35^. The transition from high-valence Fe to low-valence Fe by GSH was further verified by the Fe *2p* XPS spectra shifted from 710.62 eV to 709.76 eV (Supplementary Fig. 36). The single atomic Fe/CDs@PPSNs exhibited higher catalytic activity than that of iron oxide nanoparticles at the same Fe concentration (IOs/CDs@PPSNs) in the silica pore channels (Supplementary Fig. 37). Moreover, the calculated TOF values of Fe/CDs@PPSNs were much higher, suggesting its faster reaction rates and kinetics (Supplementary Tables 8 and 9), due to the most sufficient size effect and maximum surface-active sites exposure of the single-atom structure^36^. In addition, Fe/CDs@PPSNs also exhibit peroxidase (POD)-like mimic activity under acidic condition (pH=5.0) (Supplementary Fig. 38)^19^. These demonstrate that Fe/CDs@PPSNs can serve as an efficient catalyst candidate for safe tumor-specific therapy, especially for the biocatalytic cascades and selectivity operating in these confined nano/microenvironments.

### The therapeutic effect of Fe/CDs@PPSNs against tumor cells

Based on the efficient energy conversion capability (PTT and unique catalytic effects) of silica-based Fe single atoms system in the confined mesoporous network, the NIR laser-triggered ablation on SMMC-7721 (human hepatocellular carcinoma) tumor cell line by Fe/CDs@PPSNs was conducted. Firstly, confocal laser scanning microscopy (CLSM) verified the successful and effective internalization of the fluorescein isothiocyanate (FITC)-conjugated Fe/CDs@PPSNs by SMMC-7721 cells (Supplementary Fig. 39). As shown in Fig. 5e and Supplementary Fig. 40, Fe/CDs@PPSNs possess good biocompatibility toward SMMC-7721 and normal NIH-3T3 (mouse embryonic fibroblast) cells even at high particle concentration up to 1000 ppm. With the addition of 100 µM H_2_O_2_, a slight decrease of cell viability is presented, indicating the tumor catalytic toxicity of Fe/CDs@PPSNs. Under the NIR laser exposure, the cell viability of Fe/CDs@PPSNs significantly dropped to 12%, which is much lower than those of CDs@PPSNs and IOs@PPSNs (Supplementary Fig. 41). The flow cytometry analyses also reveal a highest apoptosis rate (Q2 + Q3: 79.19%) by Fe/CDs@PPSNs compared to the control groups (Fig. 5f and Supplementary Fig. 42), which can be assigned to the combined effect between catalytic therapy and PTT. In addition, an enhanced green fluorescence from the ROS detector (2′,7′-dichlorodihydrofluorescein diacetate (DCFH-DA)) can be clearly observed in cells treated with H_2_O_2_ and Fe/CDs@PPSNs (Fig. 5g). The results serve the great potentials of Fe/CDs@PPSNs with highly active Fe single atoms for the catalytic therapy by the high-efficient ROS generation. Besides, in the live/dead cell staining test as shown in Fig. 5h, all the control group (I), NIR laser group (II), CDs@PPSNs (III), and Fe/CDs@PPSNs group (V) display strong green fluorescence (living cells), revealing the negligible or insignificant cell death due to the insufficient ROS production or moderate temperature increase. After NIR irradiation, SMMC-7721 cells treated with Fe/CDs@PPSNs + H_2_O_2_ + NIR (VIII) show intense red fluorescence (dead cells), which is consistent with the cell viability and ROS staining tests, verifying the safe and efficient tumor therapeutic application of single atomic Fe/CDs@PPSNs.

### Therapeutic effects and bio-distribution of Fe/CDs@PPSNs in vivo

**Fig. 6:**
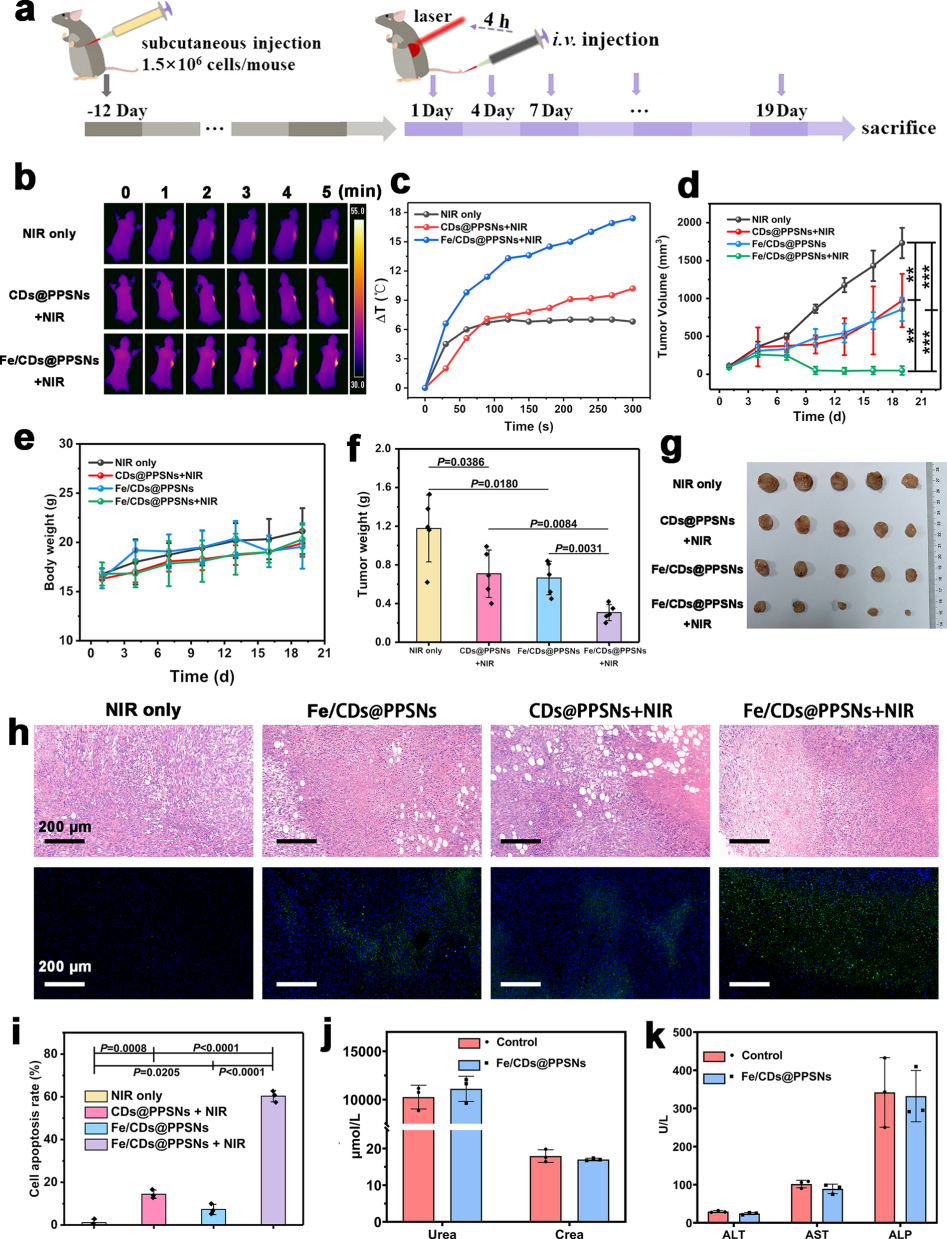
Therapeutic effects of Fe/CDs@PPSNs in vivo. **a** Establishment of SMMC-7721 tumor models and experimental procedures for the combined PTT and catalytic therapy. **b** In vivo infrared thermal imaging images and (**c**) temperature increase curves of SMMC-7721 tumors-bearing mice treated with PBS, CDs@PPSNs, and Fe/CDs@PPSNs upon 808 nm laser irradiation. **d** Tumor proliferation and (**e**) body weight curves of mice after intravenous administration with different treatment. **f** Average tumor weights of different groups after the whole treatment. **g** Digital photographs of dissected tumors from different groups. **h** H&E (upper) and TUNEL (lower) staining pictures of tumor tissues collected from different groups of mice. **i** Cell apoptosis rate measured by TUNEL staining. **j** Blood biochemistry assays of kidney function markers: urea and creatinine (Crea). **k** Blood biochemistry assays of liver function markers: alanine aminotransferase (ALT), aspartate aminotransferase (AST), and alkaline phosphatase (ALP). The data are expressed as means ± s. d. from five (**d**, **e**, **f**) or three (**i**, **j**, **k**) independent replicates. *p* values were analyzed by Student’s two-sided t-test (*p < 0.05, **p < 0.01, ***p < 0.001).

Encouraged by the satisfactory photothermal effect and the Fenton catalytic activity in vitro, the in vivo therapeutic effects of Fe/CDs@PPSNs were evaluated based on the SMMC-7721 tumor-bearing mouse model (Fig. 6a). Firstly, the bio-distribution of Fe/CDs@PPSNs in vivo was evaluated by the indocyanine green (ICG) loaded agents with the hydrodynamic diameter of ~295 nm and zeta potential of -8.33 ± 0.26 mV (ICG@PPSNs, Supplementary Fig. 43). As shown in Supplementary Fig. 44a, b, compared to the free ICG group, ICG@PPSNs group exhibits stronger fluorescence signals after 1 h injection due to the stabilization of silica matrix and shows the strongest fluorescence in tumor sites at 4 h post-injection. Besides, the tumors and major organs of mice treated with pure ICG and ICG@PPSNs were harvested and imaged ex vivo (Supplementary Fig. 44c). The tumor in the ICG@PPSNs-treated group still maintains a recognizable fluorescence signal at 24 h post-injection, indicating the good retention ability of ICG@PPSNs in tumor sites (Supplementary Fig. 44d). To acquire more accurate quantitative data on the in vivo bio-distribution, inductively coupled plasma atomic emission spectrometry (ICP-AES) was employed by quantitatively analyzing the Si contents in the tumor and various organs. The bio-distribution assay (Supplementary Fig. 44e) exhibits that the amount of Si elements in the tumor tissues reached a maximum value at 4 h post-injection of Fe/CDs@PPSNs and then decreased with the prolonged time to 24 h. Furthermore, the content of Si element in liver, spleen and kidney was detected, which can be attributed to the active uptake of the reticuloendothelial (RES) system. Besides, it is noted that the concentration of Si in heart was still high, which may be attributed to the residual blood in heart organs. To better understand the in vivo bio-distribution, the total percentages in tumors and various organs were calculated. As shown in Supplementary Fig. 44f, 24.4% of Si was found in liver at 4 h, while the amount of Si element in the tumor is about 13.8% at 24 h, demonstrating the good accumulation of Fe/CDs@PPSNs in tumor region. Consequently, both NIR fluorescence imaging and ICP quantitate results show that Fe/CDs@PPSNs can not only accumulate efficiently in tumor sites of mice but also can metabolize out by liver and kidney.

As displayed by thermal imaging and time-temperature curves (Fig. 6b, c), the local tumor temperature of mice treated with Fe/CDs@PPSNs was increased by 17.4 °C within only 5 min upon the irradiation of an 808 nm laser, which is high enough to kill tumor cells. As observed in Fig. 6d, tumor-bearing mice treated with laser alone show no significant inhibition effect on tumor growth under this safe power. Noticeably, Fe/CDs@PPSNs show a certain tumor inhibition effect, demonstrating the effectiveness of catalytic therapy-mediated antitumor efficacy. And the tumor growth suppression effect of Fe/CDs@PPSNs was largely enhanced by 808 nm laser irradiation, mainly owing to the collaborative effect between PTT and catalytic therapy. Moreover, no erratic fluctuations in the body weight of all groups were observed (Fig. 6e), revealing the negligible side effects caused by the treatments. The tumor weights and digital photographs of sliced tumor tissues from mice on the 19th day further confirm the highest tumor inhibition effect of Fe/CDs@PPSNs plus 808 nm laser irradiation in vivo (Fig. 6f, g). Additionally, obvious tumor cell death of Fe/CDs@PPSNs + 808 nm group could be observed from the hematoxylin-eosin (H&E) and TdT-mediated dUTP nick-end labeling (TUNEL) immunofluorescence images (Fig. 6h), with the highest cell apoptosis rate (60.7%) measured by TUNEL staining image (Fig. 6i).

To ensure the biosafety of the Fe/CDs@PPSNs for potential practical applications, healthy Kunming mice were intravenously injected with Fe/CDs@PPSNs, and then the blood samples from Kunming mice were collected for serum chemistry and hematological analyses. As shown in Fig. 6j, k, no visible hepatic or renal toxicity can be observed 12-day post intravenous injection of Fe/CDs@PPSNs. Besides, all the hematological biomarkers show no distinct abnormalities after the injections for 12 days (Supplementary Table 10), indicating the negligible side effects of Fe/CDs@PPSNs on the hematological system. Finally, the major organs (hearts, livers, spleens, lungs, and kidneys) of mice were collected on the 19th day for hematoxylin-eosin (H&E) staining (Supplementary Fig. 45). These hematology analyses show the satisfactory biosafety of Fe/CDs@PPSNs.

In summary, we have developed a facile and efficient interfacial-confined coordination strategy for the construction of Fe single-atom nanotherapeutic agents to achieve safe tumor-specific therapy. Such a nanotherapeutic agent was prepared by a confined carbonization process in the pore channels of mesoporous silica nanoparticles, which converts the intra-pore distributed polymeric templates (e.g. PS-*b*-PAA and CTAB) and oleylamine capping agents to nitrogen-doped carbon dots, and subsequently induce the interfacial chemical coordination between nitrogen and Fe atoms confined at the interface between nitrogen-doped carbon dots and iron oxide nanoparticles, forming the Fe-N-C single atom structure in biocompatible porous silica nanoreactor. The resulting Fe/CDs@PPSNs containing single atom Fe-N-C Fenton catalyst not only possess excellent photothermal effect under exogenous NIR irradiation through a Fe single atoms-anchored defective carbon-mediated mechanism, but also exhibit a tumor-specific, dual pH/GSH-responsive Fenton catalytic activity for hydroxyl radical generation catalyzed by the Fe single atoms. More importantly, as a proof of concept, the biological effects on the combined photothermal and catalytic therapeutic modality of Fe/CDs@PPSNs without significant toxicity to normal tissues/organs have been demonstrated both in vitro and in vivo. This work may open a new avenue to develop high-quality single-atom nanotherapeutic agents of varied components and catalytic activities for the safe and efficient tumor-specific therapy.

## Methods

### Materials

Hexadecyltrimethylammonium bromide (CTAB), tetrahydrofuran (THF), Fluorescein isthiocyanate (FITC), ammonium hydroxide (NH_3_·H_2_O), and 3-aminopropyl triethoxysilane (APTES) were all purchased from Aladdin. Ltd. Hydrochloric acid (HCl) and tetraethyl orthosilicate (TEOS) were obtained from Lingfeng Chemical Reagent Co. Ltd and ethanol (AR) was purchased from Titan (Shanghai, China). mPEG-Silane (MW 5000 Da) were purchased from Sigma-Aldrich (Natick, MA, USA). Cell-related agents were all obtained from KeyGEN BioTECH (Nanjing, China). All chemicals were used as received without further purification.

### Characterization

The high-angle annular dark field scanning transmission electron microscopy (HAADF-STEM) images were recorded by a JEOL JEM-ARM200F STEM with a spherical aberration corrector working at 300 kV. Transmission electron microscopy (TEM) was performed on a Thermo Fisher Themis Z microscope equipped with two aberration correctors under 300 kV. High angle annular dark field (HAADF)-STEM images were recorded using a convergence semi angle of 11 mrad, and inner- and outer collection angles of 59 and 200 mrad, respectively. Energy dispersive X-ray spectroscopy (EDS) was carried out using 4 in-column Super-X detectors. Dynamic light scattering (DLS) experiments were performed on a NanoZS (Malvern Instruments, Malvern, UK) at 25 °C. N_2_ absorption/desorption isotherms were obtained on a Micromeritics Tristar II 3020 system. Fe concentration was measured by inductively coupled plasma atomic emission spectroscopy (ICP-AES). Carbon content was tested with a VARIO EL III elemental analyzer. X-ray photoelectron spectroscopy was carried out by Thermo ESCALAB 250Xi. Confocal microscopy images were taken using a FV1000 Confocal Microscope (Olympus). Flow cytometry was performed by BD Accuri C6.

XAFS measurements at the Fe K-edge were performed at the BL14W1 in Shanghai Synchrotron Radiation Facility (SSRF). The electron beam energy was 3.5 GeV, and the stored current was 260 mA (top-up) using a double-crystal Si (111) monochromator. The Fe/CDs@PPSNs samples were recorded under fluorescence mode while the standard Fe-foil and Fe_2_O_3_ samples were recorded under transmission mode. The acquired spectra of Fe/CDs@PPSNs, Fe-foil, and Fe_2_O_3_ were calibrated and processed by Athena program in IFEFFIT software (version: 1.2.11d) package. The Fourier transform (FT) of Fe K-edge EXAFS spectra in k-space and R-space of the samples were performed with a k-weighting number of 3 under proper widths of Hanning window functions. These spectra were then fitted according to the FEFF theory based on the first-shell approximation in Artemis software (version: 0.9.26) using Fe-O, Fe-Fe, and Fe-C scattering paths.

### Synthesis of Fe/CDs@PPSNs

In all, 6 nm OA-stabilized Fe_3_O_4_ NPs were prepared through thermal decomposition of Fe (acac)_3_ at 300 °C^23^. Amphiphilic block copolymer PS_96_-*b*-PAA_16_ was synthesized via sequential atomic transfer radical polymerization (ATRP)^37^. In a typical synthesis, 50 mg PS_96_-*b*-PAA_16_ was first dissolved in 10 mL of THF containing 25 mg OA-stabilized Fe_3_O_4_, then poured into a solution including 40 mL water, 100 mg CTAB, and 0.5 mL NH_3_·H_2_O. The resulting mixture was then poured into 80 mL ethanol containing 0.3 g TEOS. After stirring for 10 min, the solution was allowed to stand at 35 °C for 18 h. The products were then collected by centrifugation, washed with ethanol twice, further dried in a vacuum oven at 35 °C. The carbonization was conducted in a tube furnace with nitrogen as the fluidizing gas at different temperatures, resulting in the formation of iron oxide/carbon dots co-loaded porous silica nanoparticles (designated as IOs/CDs@PSNs). To remove the iron oxide nanoparticles, the obtained IOs/CDs@PSNs was stirred with a 1 M HCl solution at 30 °C for 24 h, the process was repeated for three times and then washed with water. Finally, mPEG-Silane (Mw = 5000) was added to the above solution and stirred for 24 h at room temperature. The final product of Fe/CDs@PPSNs was obtained after three successive washes with pure water. For comparison, IOs@PPSNs were obtained by calcining in air atmosphere and CDs@PPSNs were prepared using the same procedure as described above without adding Fe_3_O_4_. In addition, IOs/CDs were obtained with the same procedure of IOs/CDs@PSNs except for the addition of TEOS, and Fe/CDs are prepared by stirring with a 1 M HCl solution for 24 h, this process was repeated three times.

### Synthesis of Ni/CDs@PPSNs and Mn/CDs@PPSNs

Hydrophobic Ni nanoparticles were prepared by the reduction of nickel (II) acetylacetonate with the borane-tributylamine complex in a mixture of oleylamine and oleic acid^38^. Mn_3_O_4_ NPs were obtained by decomposition of Mn(acac)_2_ at 180 °C^39^. Then, Ni/CDs@PPSNs and Mn/CDs@PPSNs were prepared by the in-situ carbonization of Ni@PSNs and Mn_3_O_4_@PSNs precursor and the subsequent acid etching, which is similar to the synthesis of Fe/CDs@PPSNs except for using Ni or Mn_3_O_4_ instead of Fe_3_O_4_.

### Computational details

DFT calculations were performed using the Vienna ab initio simulation package (VASP)^40,41^. The projector augmented wave (PAW) method was employed to describe the interactions between ion cores and valence electrons^42^. The GGA-PBE was used to describe the exchange-correlation functional^43^. The solution of the Kohn-Sham equations was expanded in a plane wave basis set with a cutoff energy of 400 eV. The Brillouin zone sampling was performed using a Monkhorst-Pack grid^44^, and electronic occupancies were determined in light of a Gaussian smearing with a width of 0.05 eV. In all the calculations, a force-based conjugated gradient method was used to optimize the geometries^45^. All of the graphene models are constructed based on the graphene basal plane model with the supercell of $5\times 3\sqrt{3}\times 1$ (12.30 Å × 12.78 Å × 15.00 Å), with a relaxation of all atoms. The Brillouin-zone integration has been performed with a 3 × 3 × 1 Monkhorst-Pack k-point mesh. The geometry optimizations were considered to be converged when the maximum force in each degree of freedom was <0.03 eV Å^−1^. Bader charge analysis was implemented with a fast algorithm developed by Henkelman and coworkers, and the core charges were included in the partitions^46,47^ The charge density difference images were obtained by the VESTA visualization software. The charge density difference (Δ*ρ*) isosurface is calculated by Δ*ρ*(r) = *ρ*_FeN4/C_(r)−*ρ*_Fe_(r)−*ρ*_N4/C_ for the FeN_4_/C model and Δ*ρ*(r)  = *ρ*_N4C_(r)−*ρ*_N_(r)−*ρ*_C_ for the N_4_C model, where the *ρ*(r) is the electron density.

### The Fe release experiments

The Fe release experiments from Fe/CDs@PPSNs and IOs/CDs@PPSNs were conducted by dialysis solution assay as below: 1 mg of Fe/CDs@PPSNs or IOs/CDs@PPSNs were dispersed in 4 ml PBS (10 mM, pH = 5.0 or 7.4) and transferred into a dialysis bag (cut off = 14 kDa). Then, it was immersed in 12 ml PBS and incubated at a 37 °C shaker with a rotating speed of 140 rpm. At given time points of 1 h, 4 h, 8 h, 12 h, 24 h, 48 h, 6 mL PBS was taken out and analyzed by ICP-AES. To maintain the constant volume, 6 ml of fresh PBS was added into the initial releasing medium for further test.

### In vitro photothermal experiments

The aqueous suspensions at varied concentrations (1 mL) in quartz cuvette were irradiated with 808 nm laser (Hps3200, B&A Technology Co., Ltd) at varied power for 5 min. The temperature of the solutions was recorded using an infrared thermal imaging camera (Fotric 225RD-L39). The photothermal stability of the Fe/CDs@PPSNs was conducted by irradiating with an 808 nm NIR laser for 5 min (LASER ON), followed by a natural cooling without NIR laser irradiation for 5 min (LASER OFF). This cycle was repeated five times.

### Calculation of the photothermal conversion efficiency

Calculation of the Extinction Coefficient. The extinction coefficient *ɛ*(λ) of Fe/CDs@PPSNs to evaluate the NIR absorption capability is determined according to the Lambert-Beer law

1 $${{A}}({\lambda})={\varepsilon} LC$$

*A* is the absorbance at the wavelength (λ), *L* is the pathlength (1 cm), and *C* is the concentration of the NPs (in g L^−1^). The extinction coefficient ɛ is calculated by plotting the slope (in L g^−1^ cm^−1^) of each linear fit against wavelength. The 808 nm laser extinction coefficient (*ɛ*) can be determined to be 0.25998, 0.65432, 0.6771, and 0.18303 L g^−1^ cm^−1^ for Fe/CDs@PPSNs-350, Fe/CDs@PPSNs-550, Fe/CDs@PPSNs, and CDs@PPSNs, respectively.

Calculation of the Photothermal Conversion Efficiency. Following the report of Roper et al.^29^, the energy balance for the system is

2 $$\mathop{\sum}\limits_{i}{m}_{i}{C}_{{{p}},{{i}}}\frac{{{dT}}}{{{dt}}}={Q}_{{{{{{\rm{Fe}}}}}}/{{{{{\rm{CDs}}}}}}@{{{{{\rm{PPSNs}}}}}}}+{Q}_{0}-{Q}_{{{{{{\rm{surr}}}}}}}$$

where *C*_p_ and m are the heat capacity and the mass of solvent (water), *T* is the solution temperature, respectively.

3 $${Q}_{{{{{{\rm{Fe}}}}}}/{{{{{\rm{CDs}}}}}}@{{{{{\rm{PPSNs}}}}}}}={{I}}(1-{10}^{-{A}_{808}}){{\eta}}$$

Specially, *I* represent the incident power, *A*_*λ*_ is the absorbance of the NPs at 808 nm given by Beer-Lambert’s Law, and *η* is the photothermal-conversion efficiency.

When the temperature of system reaches a maximum, the heat output is equal to heat input

4 $${Q}_{{{{{{\rm{Fe}}}}}}/{{{{{\rm{CDs}}}}}}@{{{{{\rm{PPSNs}}}}}}}+{Q}_{0}={Q}_{{{{{{\rm{Surr}}}}}}-{{{{{\rm{Max}}}}}}}={hS}\varDelta {T}_{{{{{{\rm{Max}}}}}}}$$

The photothermal conversion efficiency (*η*) can be obtained by substituting Eq. 3 for *Q*_Fe/CDs@PPSNs_ into Eq. 4 and rearranging to get

5 $${{\eta }}=\frac{{{hS}}({T}_{{{{{{\rm{Max}}}}}}}-{T}_{{{{{{\rm{Surr}}}}}}})-{Q}_{0}}{{{{I}}}(1-{10}^{-{A}_{808}})}$$

In order to obtain *hS*, a dimensionless driving force temperature, *θ*, is introduced, defined as follows:

6 $${{\theta }}=\frac{{{T}}-{T}_{{{{{{\rm{Surr}}}}}}}}{{T}_{{{{{{\rm{Max}}}}}}}-{T}_{{{{{{\rm{Surr}}}}}}}}$$

and a time constant for heat transfer, *τ*

7 $${{\tau }}=\frac{{\sum }_{i}{m}_{i}{C}_{{{p}},{{i}}}}{{{hS}}}$$

which is substituted into Eq. 2 and rearranged to yield

8 $$\frac{{{d}}{{\theta }}}{{{dt}}}=\frac{1}{\tau }\left[\frac{{Q}_{{{{{{\rm{Fe}}}}}}/{{{{{\rm{CDs}}}}}}@{{{{{\rm{PPSNs}}}}}}}+{Q}_{0}}{{{hS}}({T}_{{{{{{\rm{Max}}}}}}}-{T}_{{{{{{\rm{Surr}}}}}}})}-{{\theta }}\right]$$

When laser radiation turned off, *Q*_materials_ + *Q*_0_ = 0, changing Eq. 8 to

9 $${{dt}}=-{{\tau }}\frac{{{d}}{{\theta }}}{\theta }$$

and integrating gives the expression

10 $${{t}}=-{{\tau }}\,{ln}\,{{\theta }}$$

Therefore, the corresponding 808 nm laser photothermal conversion efficiencies of Fe/CDs@PPSNs-350, Fe/CDs@PPSNs-550, Fe/CDs@PPSNs, and CDs@PPSNs can be calculated to be 43.8%, 56.4%, 58.1%, and 18.7% respectively.

### Hydroxyl radical generation

Detection of ·OH was using the classical colorimetric method which was based on the methylene blue (MB) degradation. In detail, Fe/CDs@PPSNs (250 µg/ml) were added into the mixture containing MB (12.5 µg/ml) and H_2_O_2_ (100 µM), for different treatment groups, the pH was adjusted to 5.0, 6.0, 7.4, respectively. In order to demonstrate the effect of GSH, different concentration of GSH was added to the system.

Electron paramagnetic spin spectrometer (ESR) spectroscopy was further performed to detect the generation of ·OH using 5,5-dimethyl-1-pyrroline N-oxide (DMPO) as a spin trapping agent. The reaction groups were mixed with 200 µl aqueous solution containing DMPO (1 mM, Aladdin) and Fe/CDs@PPSNs (250 µg mL^−1^). The mixture was transferred into a quartz capillary and recorded on a Bruker EMX-8/2.7 spectrometer.

### Preparation of FITC-grafted Fe/CDs@PPSNs

Fifty-milligram FITC was reacted with 500 µl APTES in 5 mL ethanol for 24 h in the dark^48^. Then 100 µl FITC-APTES solution and 10 mg Fe/CDs@PPSNs were added to 5 mL ethanol with shaking for overnight in the dark. Finally, the resulting product was washed with ethanol and PBS several times until the supernatant become colorless.

### Cell culture

SMMC-7721 cells and NIH-3T3 cells were purchased commercially from KeyGEN BioTECH and cultured in RPMI 1640 or DMEM supplemented with 10% fetal bovine serum at 37 °C under an atmosphere of 5% CO_2_.

### Confocal laser scanning microscopy analysis

SMMC-7721 cells (10^4^ cells per dish) were cultured in a CLSM dish at 37 °C for 24 h. After complete adhesion, the solution was replaced with fresh medium containing 100 µg mL^−1^ FITC-labeled Fe/CDs@PPSNs, then incubated for another 4 h. The cells were then washed three times with PBS and stained for 10 min with DAPI. Cells were then imaged with an excitation wavelength of 404 nm (for DAPI) and 488 nm (for FITC).

### Cytotoxicity of Fe/CDs@PPSNs

The in vitro cytotoxicity of Fe/CDs@PPSNs against SMMC-7721 cells and NIH-3T3 cells was performed by the MTT assay. In details, SMMC-7721 cells (5000 cells per well) and NIH-3T3 cells (5000 cells per well) were seeded in 96-well plates for 24 h. Then Fe/CDs@PPSNs with different particle concentrations (200, 400, 600, 800, and 1000 ppm) were added to each well and incubated for 24 h. Finally, the cytotoxicity was expressed as the percentage of cell viability as determined by the MTT assay.

### Photothermal effect for tumor cells

Cytotoxicity assay using Fe/CDs@PPSNs, CDs@PPSNs, and IOs@PPSNs combined with NIR laser irradiation was performed. SMMC-7721 cells (5000 cells per well) were firstly seeded in 96-well plates for 24 h. Then different concentrations of the three suspensions (15.625, 31.25, 62.5, 125, and 250 ppm) were added to each well and incubated for an additional 4 h. The cells were then irradiated using an NIR laser (2.0 W/cm^2^) for 5 min and incubated for an additional 20 h. Finally, the cytotoxicity was expressed as the percentage of cell viability as determined by the MTT assay.

### Evaluation of intracellular ROS generation via DCFH-DA assay

The intracellular generation of ROS was determined by a fluorogenic reagent 2,7-dichlorofluorescein diacetate (DCFH-DA), which could be oxidized to highly fluorescent DCF by ROS. SMMC-7721 cells (10^5^) with RPMI 1640 in each well were seeded and allowed to adhere at 37 °C for 24 h. After that, the growth medium was replaced with a fresh one including the following groups: 100 µM H_2_O_2_, 250 ppm Fe/CDs@PPSNs, 100 µM H_2_O_2_ with 250 ppm Fe/CDs@PPSNs and 100 µM H_2_O_2_ with 250 ppm Fe/CDs@PPSNs + 808 nm NIR laser. After co-incubation for 12 h at 37 °C, 1 ml of fresh culture media containing DCFH-DA was added to each well, and the cells were cultured at 37 °C for another 30 min. After washed with PBS for three times, the CLSM images of the samples were collected on a CLSM imaging system (*λ*ex = 488 nm, *λ*em = 525 nm).

### Calcein-AM/PI assay staining

Photothermal-induced cytotoxicity of Fe/CDs@PPSNs, IOs@PPSNs, and CDs@PPSNs was evaluated using SMMC-7721 cells. Briefly, SMMC-7721 cells (10^5^ cells per well) were incubated in a humidified atmosphere containing 5% CO_2_ at 37 °C for 24 h. Cells were then incubated with Fe/CDs@PPSNs, IOs@PPSNs, and CDs@PPSNs suspensions (1 mL per well, 250 ppm) for an additional 4 h. After treatment, cells were exposed to an NIR laser (2.0 W/cm^2^) for 5 min and stained with calcein AM (calcein acetoxythemal ester) and PI (propidium iodide).

### Animal experiments

All animal experiments were approved by Animal Ethics Committee of East China University of Science and Technology, and were conducted under the Guidance for Care and Use of Laboratory Animals of East China University of Science and Technology. All animals were housed in a specific pathogen-free condition at 26 ± 1 °C and 50 ± 5% humidity, with a 12-h light–dark cycle.

### In vivo bio-distribution

To observe the biodistribution of Fe/CDs@PPSNs in vivo, ICG molecules were encapsulated in the PPSNs by impregnation method. Then 200 µl of ICG@PPSNs (2.5 mg/ml) and ICG (200 µg/ml) was intravenously injected to SMMC-7721 tumor-bearing female mice, and their biodistribution was observed using an in vivo imaging system (IVIS^®^ Spectrum CT) at 1, 4, 8, and 24 h. Correspondingly, the tumors and organs were excised and imaged at 8 h and 24 h. To acquire more accurate quantitative data on the in vivo bio-distribution, inductively coupled plasma atomic emission spectrometry (ICP-AES) was employed by quantitatively analyzing the Si contents in the tumor and various organs. The detailed experiment was conducted as below: SMMC-7721 bearing nude mice (*n* = 3, tumor volume = 100 mm^3^) were intravenously injected with Fe/CDs@PPSNs (200 μl, 2.5 mg/ml in PBS). Then, mice were dissected at varied time intervals (1, 4, 8, and 24 h). The dissected organs and tumors were weighed and homogenized, followed by ICP-AES measurement for determining Si element concentration. The bio-distribution in different organs and tumor targeting efficiency were calculated as the percentage of injected dose per gram of tissue.

### In vivo tumor therapy and histopathological evaluation

Flank xenograft SMMC-7721 tumors were prepared by subcutaneous injection of 1.5 × 10^6^ cells into the right flanks of BALB/c female mice. The experiments were conducted when the tumor volume reached ~100 mm^3^. To evaluate the in vivo photothermal heating performance of Fe/CDs@PPSNs, IR thermal images and the temperature variation of mice were recorded at 4 h after intravenous injection of PBS, CDs@PPSNs, and Fe/CDs@PPSNs, respectively. For the tumor photothermal treatment by CDs@PPSNs and Fe/CDs@PPSNs, the mice were randomly divided into four groups (*n* = 5 per group) including: (І) PBS + NIR, (ІІ) CDs@PPSNs + NIR, (III) Fe/CDs@PPSNs, (IV) Fe/CDs@PPSNs + NIR (2.5 mg/ml). Each solution was intravenously injected into mice of a volume of 200 µl at day 1, 4, 7, 10, 13, and 16. For group І, ІІ, ІV, the tumors were irradiated by an 808 nm laser (1 W/cm^2^) for 5 min at 4 h post-injection. No further light treatments were performed. The tumor volume and body weight were measured every three days. The volume was calculated by the formula *V* = 1/2 × width^2^ × length. On day 19, all mice were euthanized, the tumors were dissected and weighted. H&E and TUNEL staining was performed for the assessment of the therapy efficiency.

### Blood biochemistry and blood routine analysis

To evaluate the biosafety of Fe/CDs@PPSNs, the hematological parameters and the blood biochemical parameters of the mice were detected after treatment with Fe/CDs@PPSNs for 12 days. The hematological parameters and the blood biochemical parameters of the mice after treatment with PBS were employed as blank controls.

### Statistical analysis

Statistical analysis was performed using a two-side Student’s *t* test and *p* < 0.05 was statistically significant differences. Digital Micrograph software, XPS peak, Jade5, Microsoft Excel 2019, Origin 2017, Flowjo_V10 were used for data processing or statistical analysis.

### Reporting summary

Further information on research design is available in the Nature Research Reporting Summary linked to this article.

## Supplementary information

Supplementary Information

Reporting Summary

## Data Availability

The experimental data supporting the findings of this study are available within the article, Supplementary Information, and Source Data. Additional data are available from the corresponding authors upon request.  Source data are provided with this paper.

## Source data

Source Data
