# Supplementary material for: Interfacial-confined coordination to single-atom nanotherapeutics
Source: Nat Commun. 2022 Jan 10;13:91. doi: 10.1038/s41467-021-27640-7 (PMC8748799; doi:10.1038/s41467-021-27640-7)
Supplement: Supplementary file 1 — Supplementary Information [file 41467_2021_27640_MOESM1_ESM.pdf]

Supplementary Information for

Interfacial-confined coordination to single-atom nanotherapeutics

Qin et al.

Contents:

Supplementary Figures 1 to 45

Supplementary Tables 1 to 10

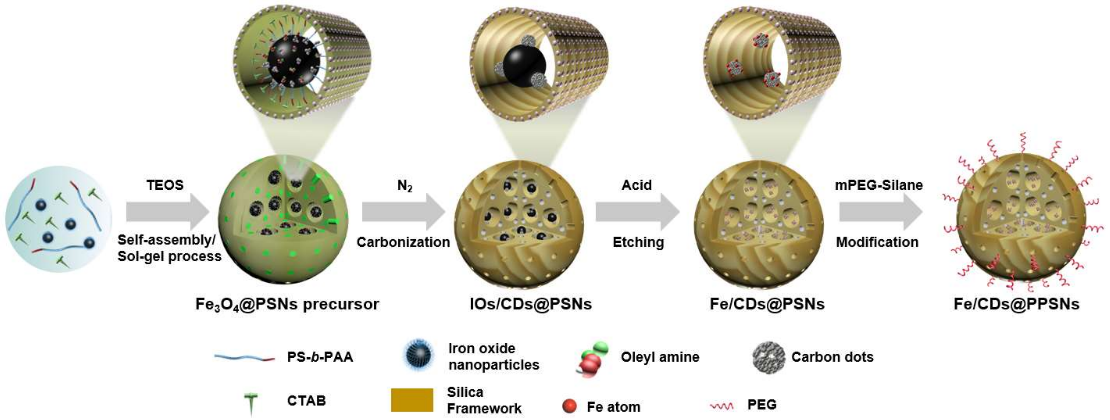

Supplementary Figure 1 Schematic diagram for the synthesis of Fe/CDs@PPSNs.

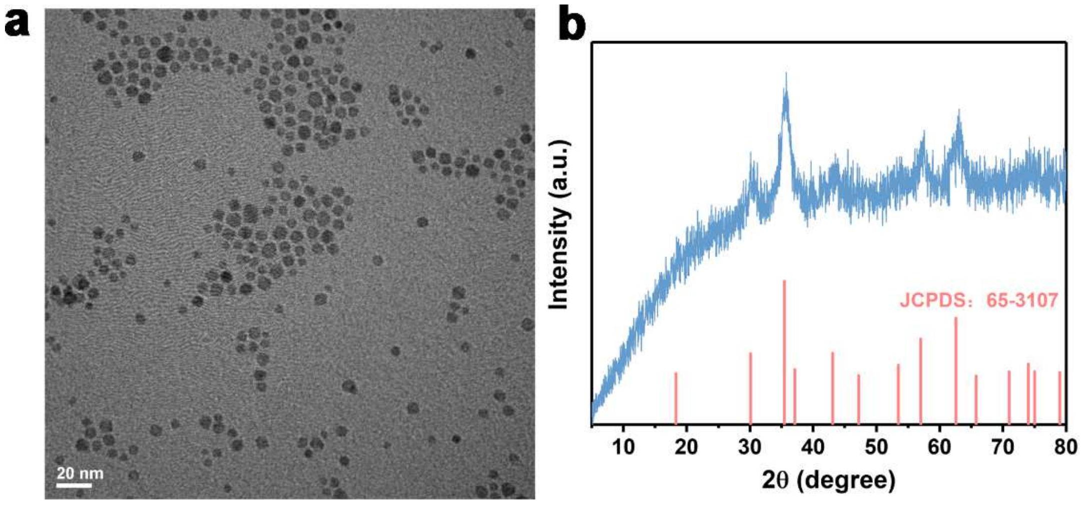

Supplementary Figure 2 **a** TEM image and **b** corresponding XRD spectrum of monodisperse Fe_3O_4 nanoparticles.

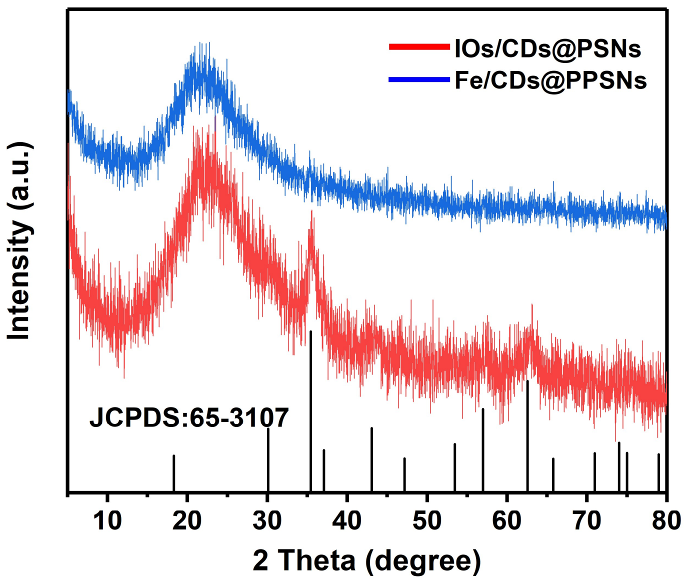

Supplementary Figure 3 Wide-angle XRD patterns of IOs/CDs@PSNs and Fe/CDs@PPSNs.

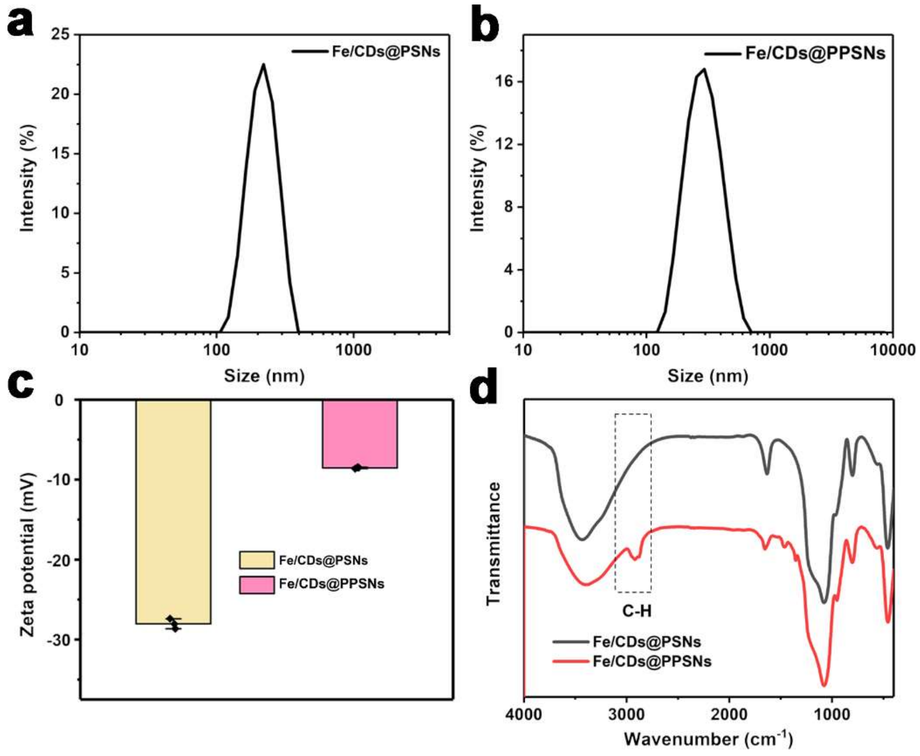

Supplementary Figure 4 Diameter distributions of Fe/CDs@PSNs before **(a)** and after **(b)** PEG modification. **c** Zeta potential and **(d)** FT-IR spectra of the Fe/CDs@PSNs before and after PEG modification. The data are expressed as means ± s. d. from three independent replicates.

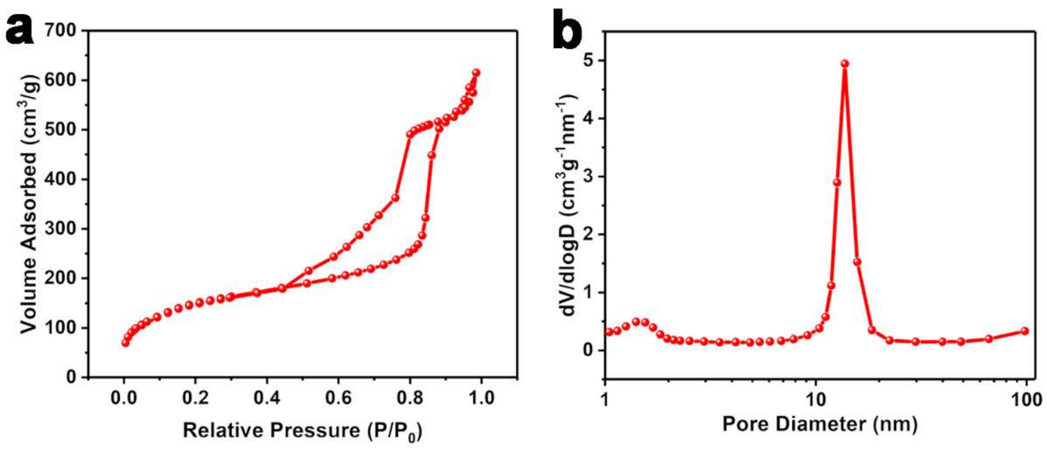

Supplementary Figure 5 N_2 adsorption/desorption isotherms **(a)** and BJH pore size distributions **(b)** of Fe/CDs@PPSNs.

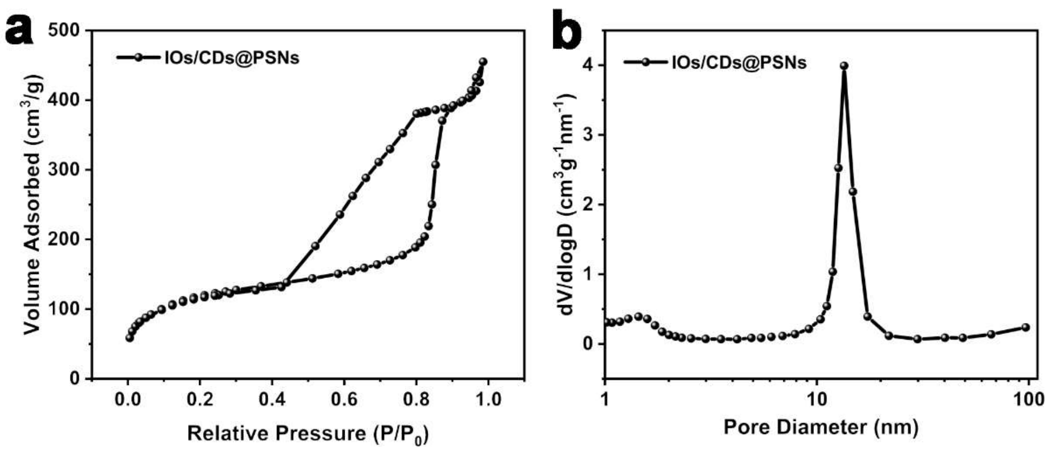

Supplementary Figure 6 (a) N_2 adsorption-desorption curve and **(b)** BJH pore size distribution of IOs/CDs@PPSNs.

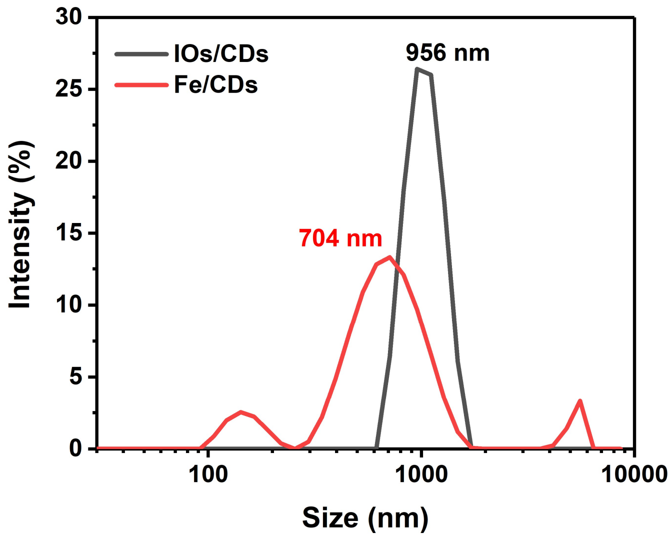

Supplementary Figure 7 Hydrodynamic diameter of IOs/CDs and Fe/CDs determined by DLS in water.

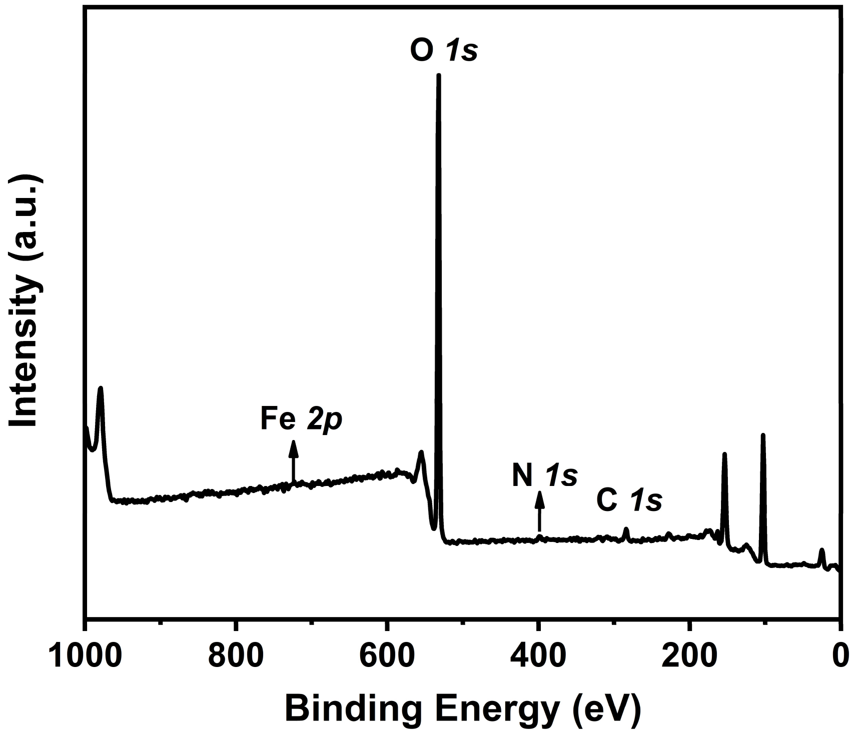

Supplementary Figure 8 Survey X-ray photoelectron spectrum (XPS) of Fe/CDs@PPSNs.

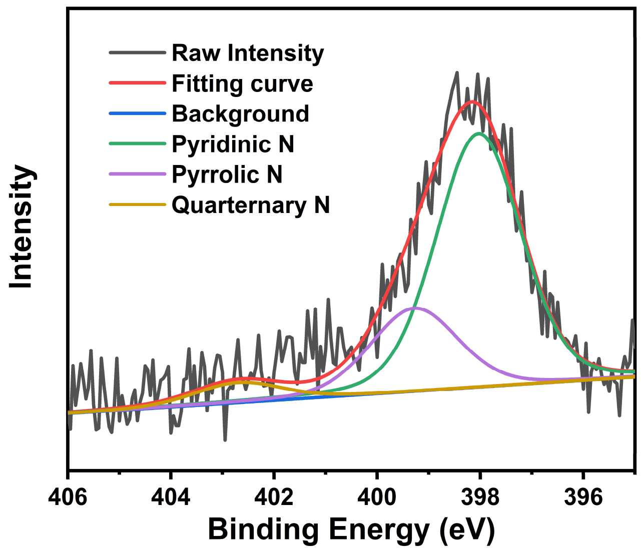

Supplementary Figure 9 N *1s* XPS spectrum of Fe/CDs@PPSNs.

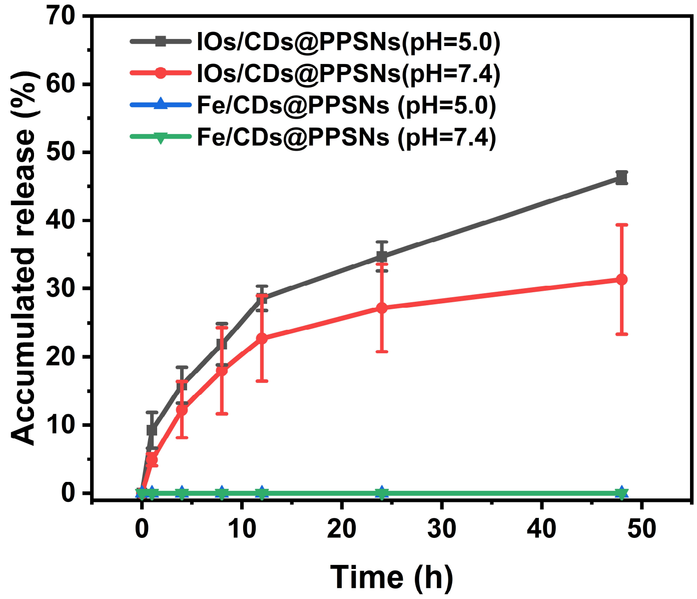

Supplementary Figure 10 The time-dependent Fe release curves from IOs/CDs@PPSNs and Fe/CDs@PPSNs under different pH conditions. The data are expressed as means ± s. d. from three independent replicates.

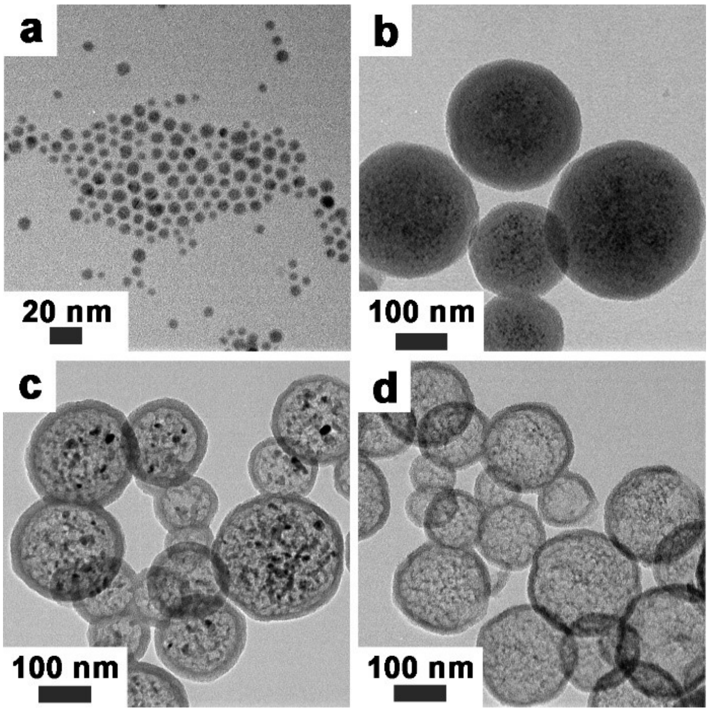

Supplementary Figure 11 TEM images of **(a)** Ni NPs, **(b)** Ni NPs@PSNs precursor, **(c)** Ni NPs/CDs@PSNs and **(d)** Ni/CDs@PPSNs.

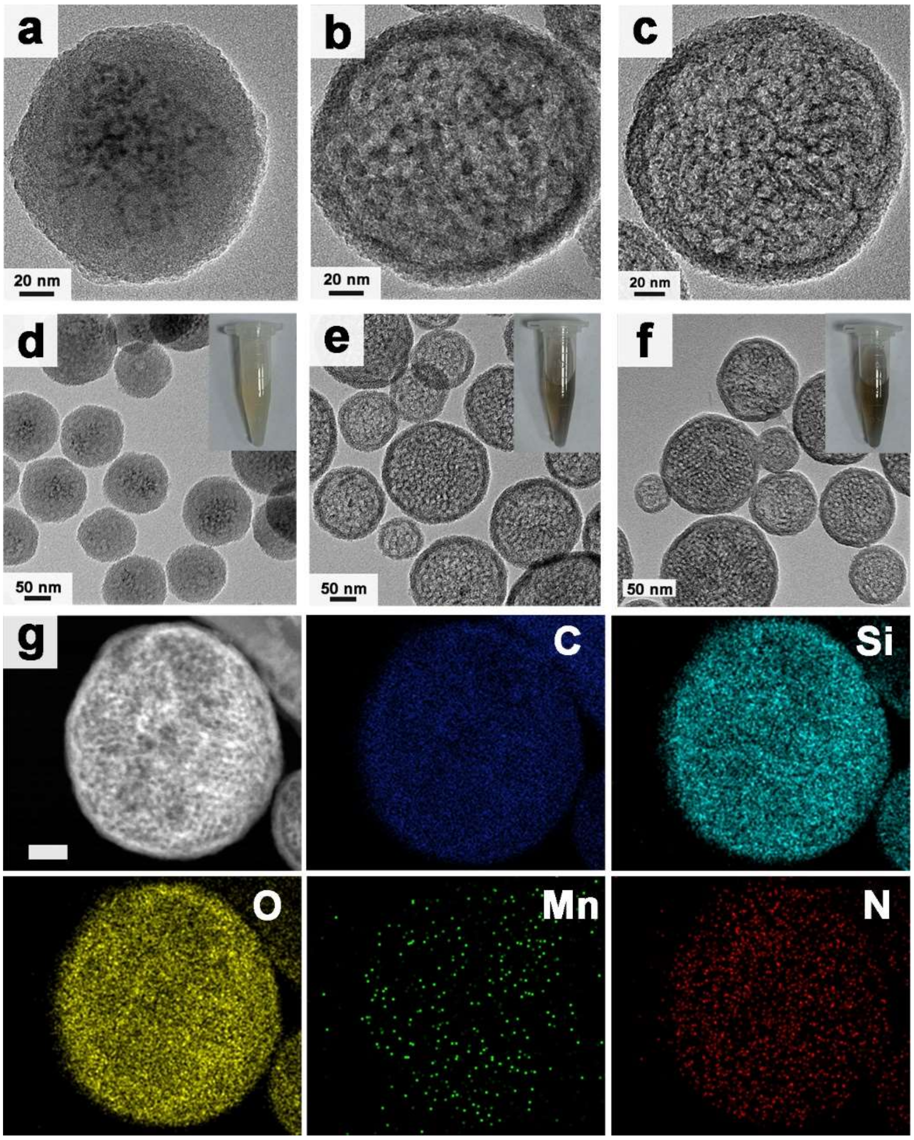

Supplementary Figure 12 Characterizations of Mn/CDs@PPSNs. TEM images and digital photographs (up-right insert) of **(a, d)** Mn_3O_4@PSNs precursor, **(b, e)** Mn_3O_4/CDs@PSNs, and **(c, f)** Mn/CDs@PPSNs. **(g)** STEM-EDS element mappings (C, Si, O, Mn, N) of Mn/CDs@PPSNs. Scale bar: 50 nm.

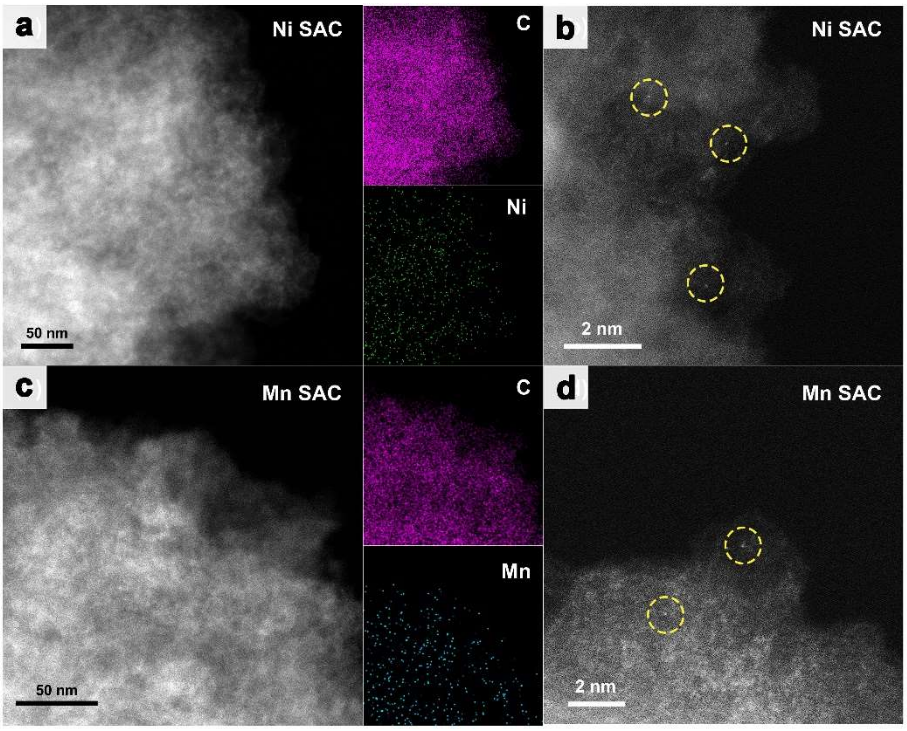

Supplementary Figure 13 Aberration-corrected HADDF-STEM images of **(a, b)** Ni/CDs@PPSNs and **(c, d)** Mn/CDs@PPSNs after the NaOH etching. The bright dots indicate the highly dispersive Ni and Mn single atoms in the carbon matrix.

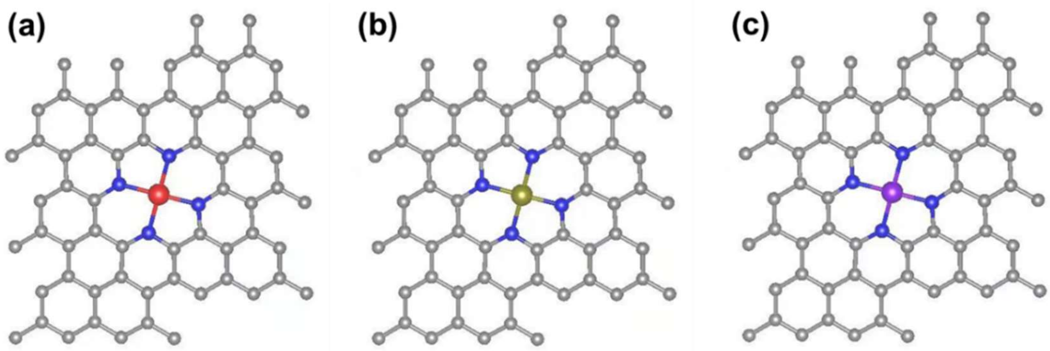

Supplementary Figure 14 The unit cells of **(a)** Fe-N_4-C, **(b)** Ni-N_4-C and **(c)** Mn-N_4-C. The gray, blue, red, yellowish green and violet balls represent the C, N, Fe, Ni and Mn atoms, respectively.

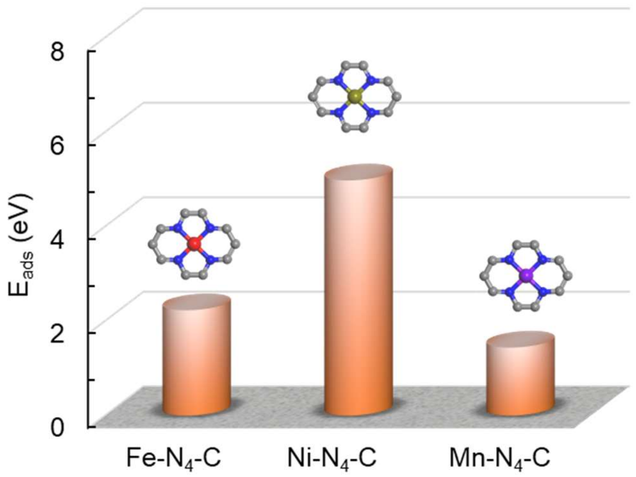

Supplementary Figure 15. Comparison for the binding energy between metal single-atom and N_4-C moiety. Insets are the configuration of M-N_4-C structures, where the red, yellowish green and violet balls denote the Fe, Ni and Mn atoms, respectively.

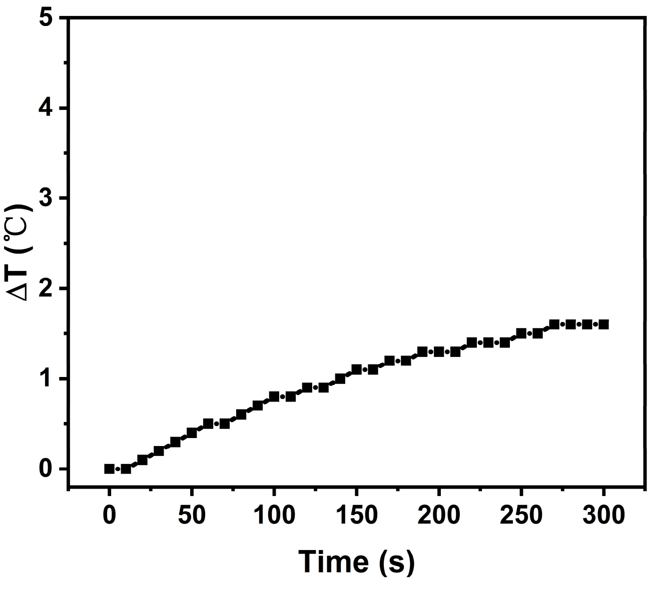

Supplementary Figure 16 The photothermal increment of water under 808 nm NIR laser at 2 W/cm^2.

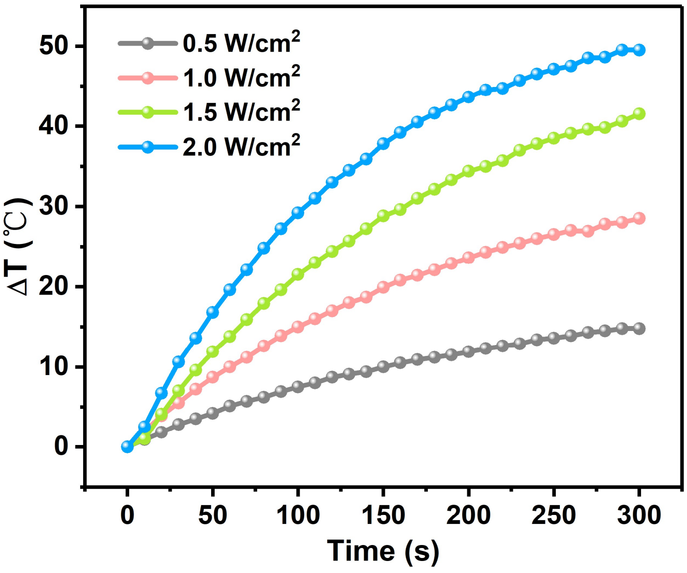

Supplementary Figure 17 Photothermal heating curves for Fe/CDs@PPSNs dispersions (0.5 mg/ml) at different laser powers (808 nm).

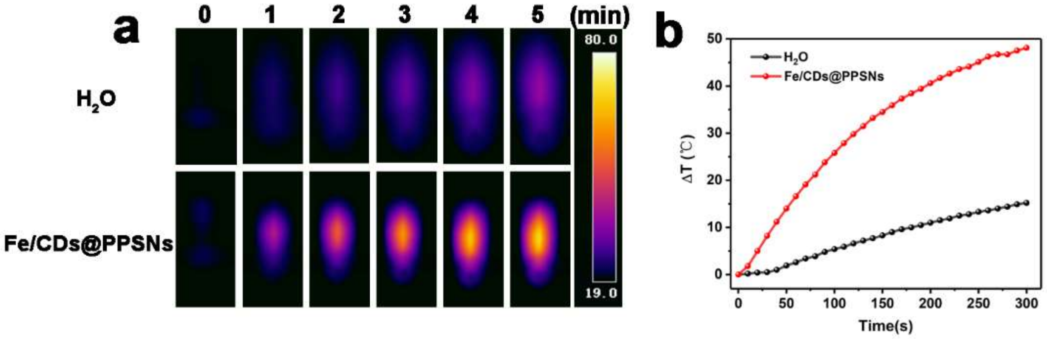

Supplementary Figure 18 (a) Infrared thermal images and **(b)** corresponding photothermal heating curves of water and Fe/CDs@PPSNs (0.5 mg/ml) with 1064 nm laser irradiation for 5 min (2 W/cm^2).

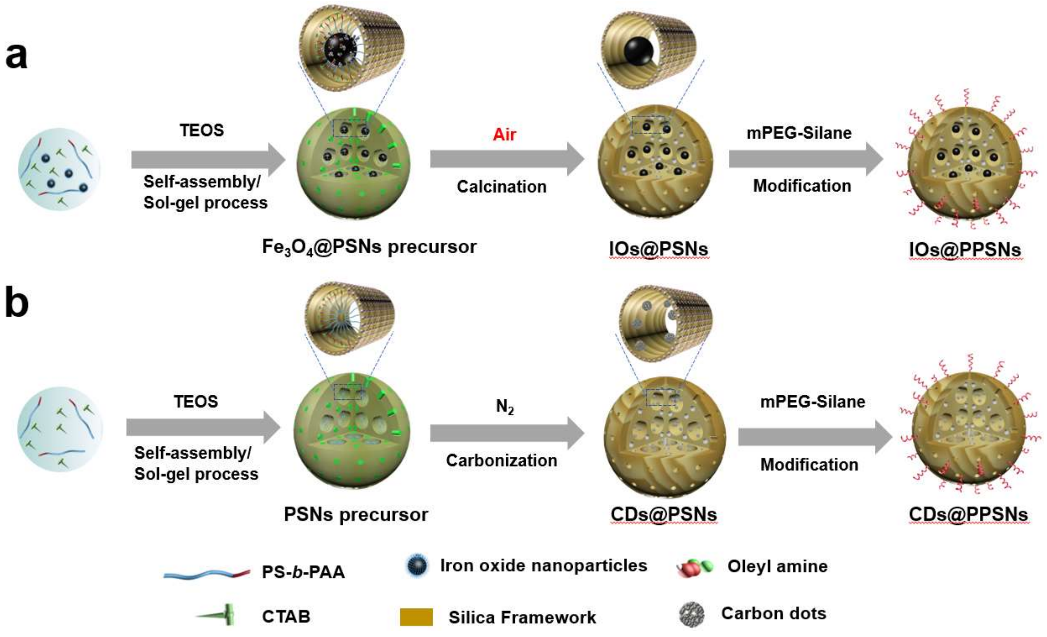

Supplementary Figure 19 Schematic diagram for the synthesis of **(a)** IOs@PPSNs and **(b)** CDs@PPSNs.

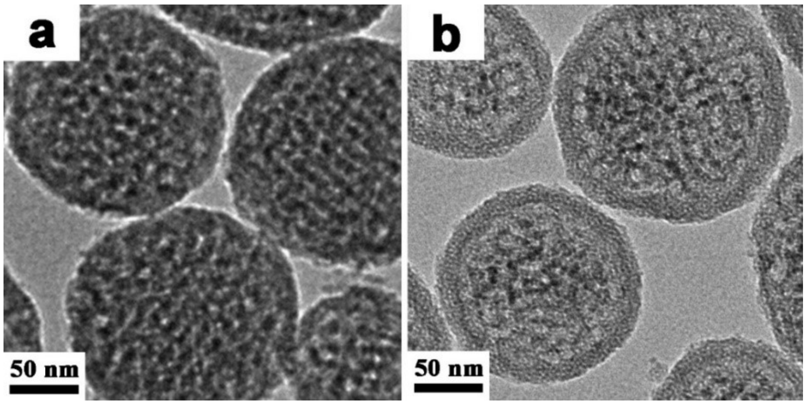

Supplementary Figure 20 TEM images of **(a)** CDs@PPSNs and **(b)** IOs@PPSNs.

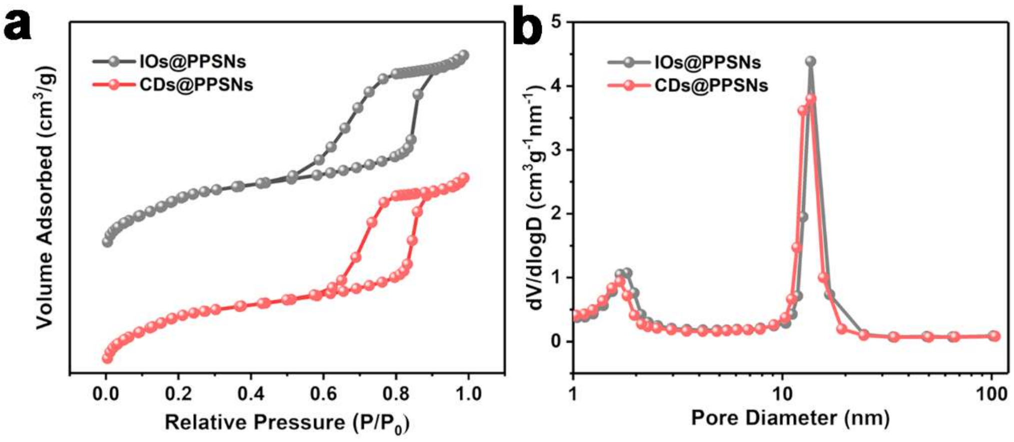

Supplementary Figure 21 (a) N_2 adsorption/desorption isotherms and **(b)** BJH pore size distributions of IOs@PPSNs and CDs@PPSNs.

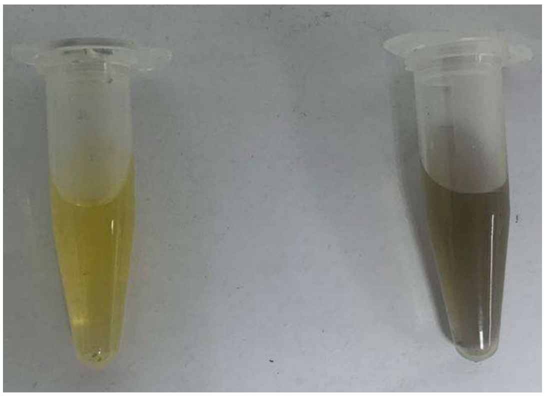

Supplementary Figure 22 Digital photographs of IOs@PPSNs (left) and CDs@PPSNs (right).

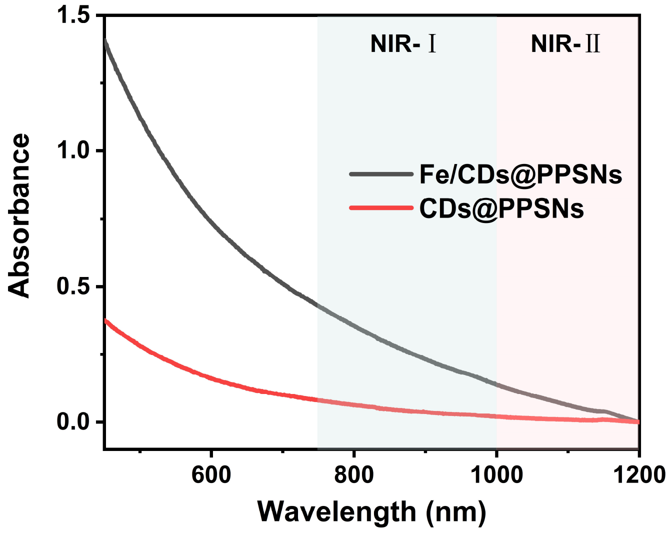

Supplementary Figure 23 UV-vis-NIR spectra of Fe/CDs@PPSNs and CDs@PPSNs at the particle concentration of 0.5 mg/mL.

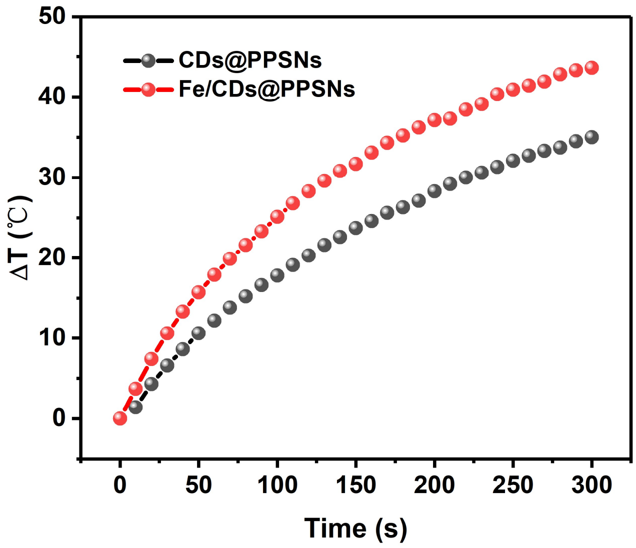

Supplementary Figure 24 Photothermal heating curves of CDs@PPSNs and Fe/CDs@PPSNs at the same carbon concentration of 15 μg/ml.

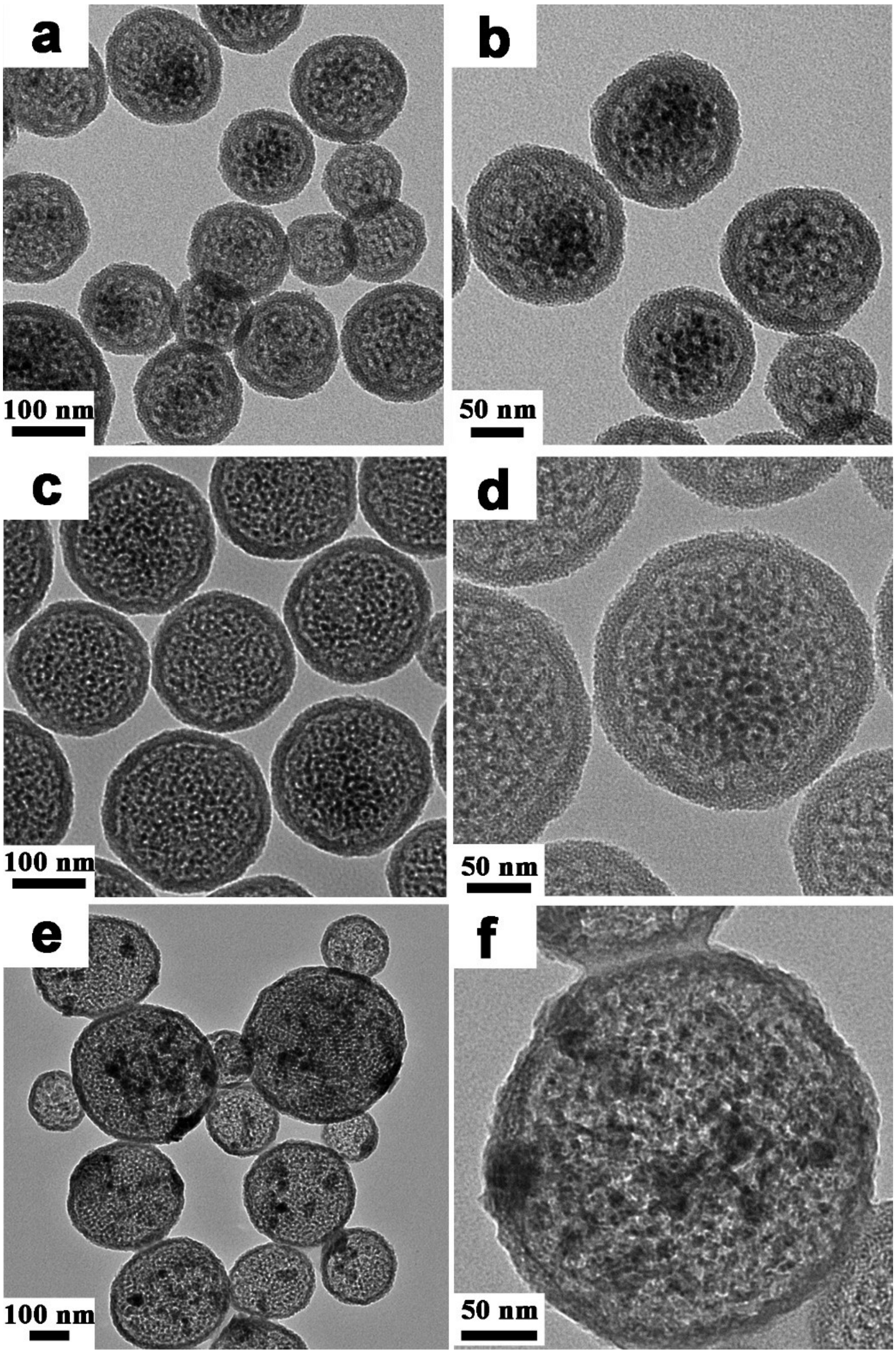

Supplementary Figure 25 TEM images of (a, b) IOs/CDs@PSNs-350, **(c, d)** IOs/CDs@PSNs-550 and **(e, f)** IOs/CDs@PSNs-800.

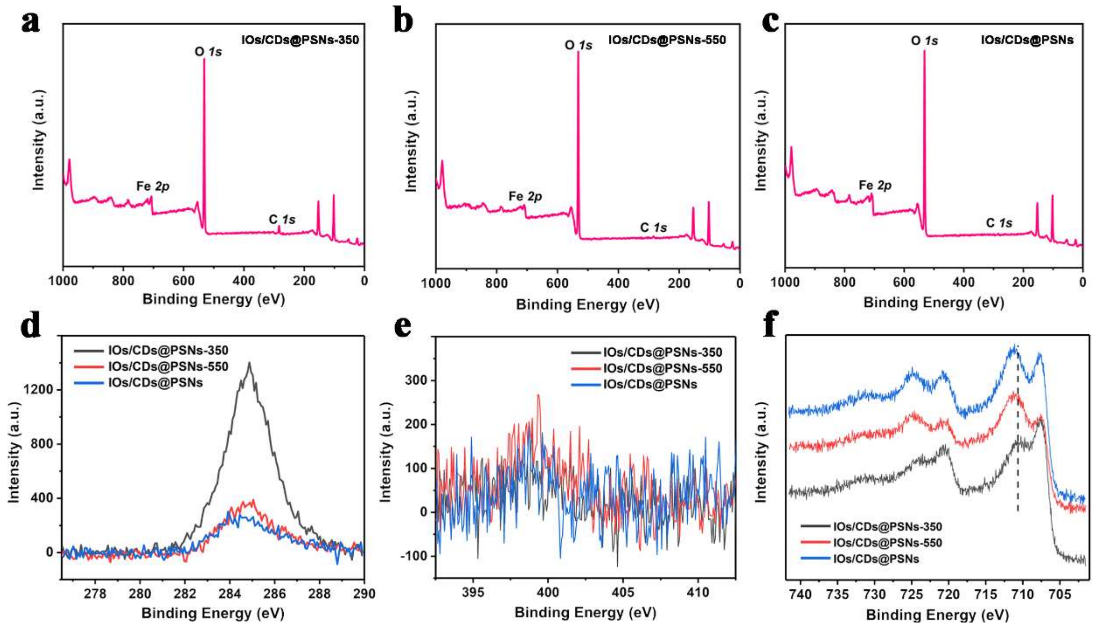

Supplementary Figure 26 Survey and fitted X-ray photoelectron spectrums (XPS) of **(a)** IOs/CDs@PSNs-350, **(b)** IOs/CDs@PSNs-550, **(c)** IOs/CDs@PSNs, **(d)** C *1s* region, **(e)** N *1s* and **(f)** Fe *2p* region.

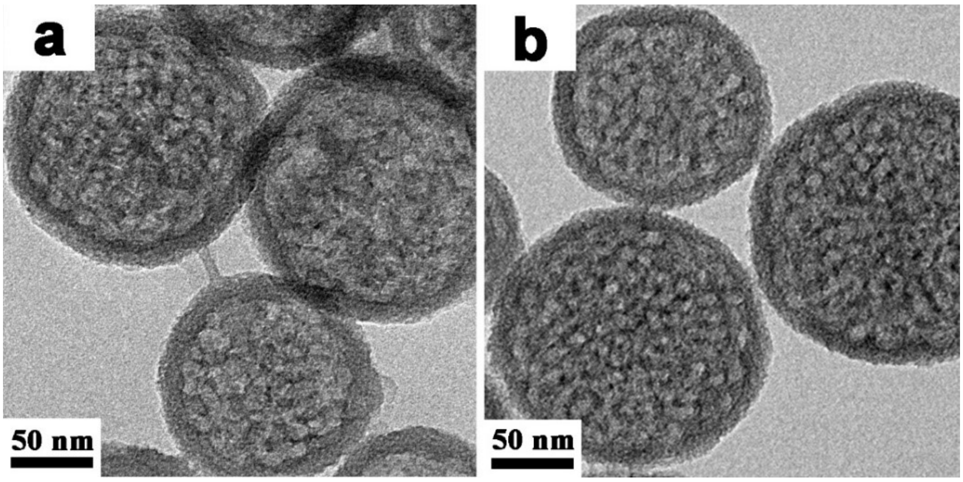

Supplementary Figure 27 TEM images of **(a)** Fe/CDs@PPSNs-350 and **(b)** Fe/CDs@PPSNs-550.

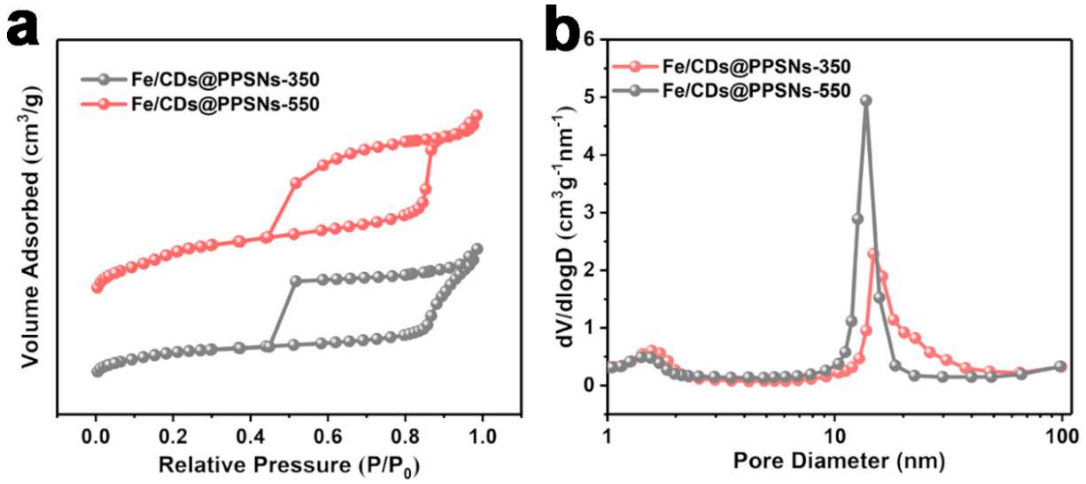

Supplementary Figure 28 (a) N_2 adsorption/desorption isotherms and **(b)** pore size distributions of Fe/CDs@PPSNs carbonized at 350°C and 550°C.

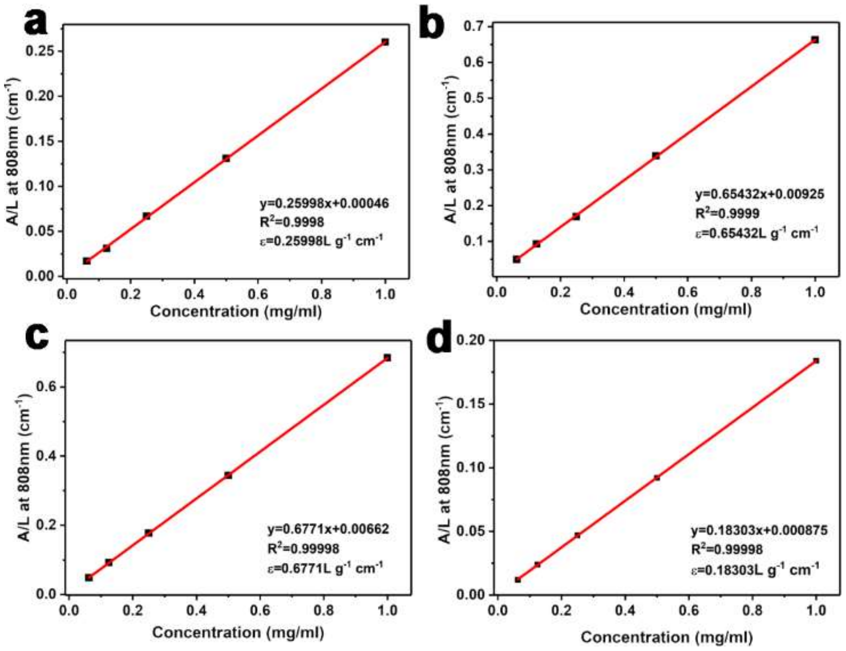

Supplementary Figure 29 Mass extinction coefficient of **(a)** Fe/CDs@PPSNs-350, **(b)** Fe/CDs@PPSNs-550, **(c)** Fe/CDs@PPSNs and **(d)** CDs@PPSNs at 808 nm. Normalized absorbance intensity at λ = 808 nm divided by the characteristic length of the cell (A/L) at varied concentrations.

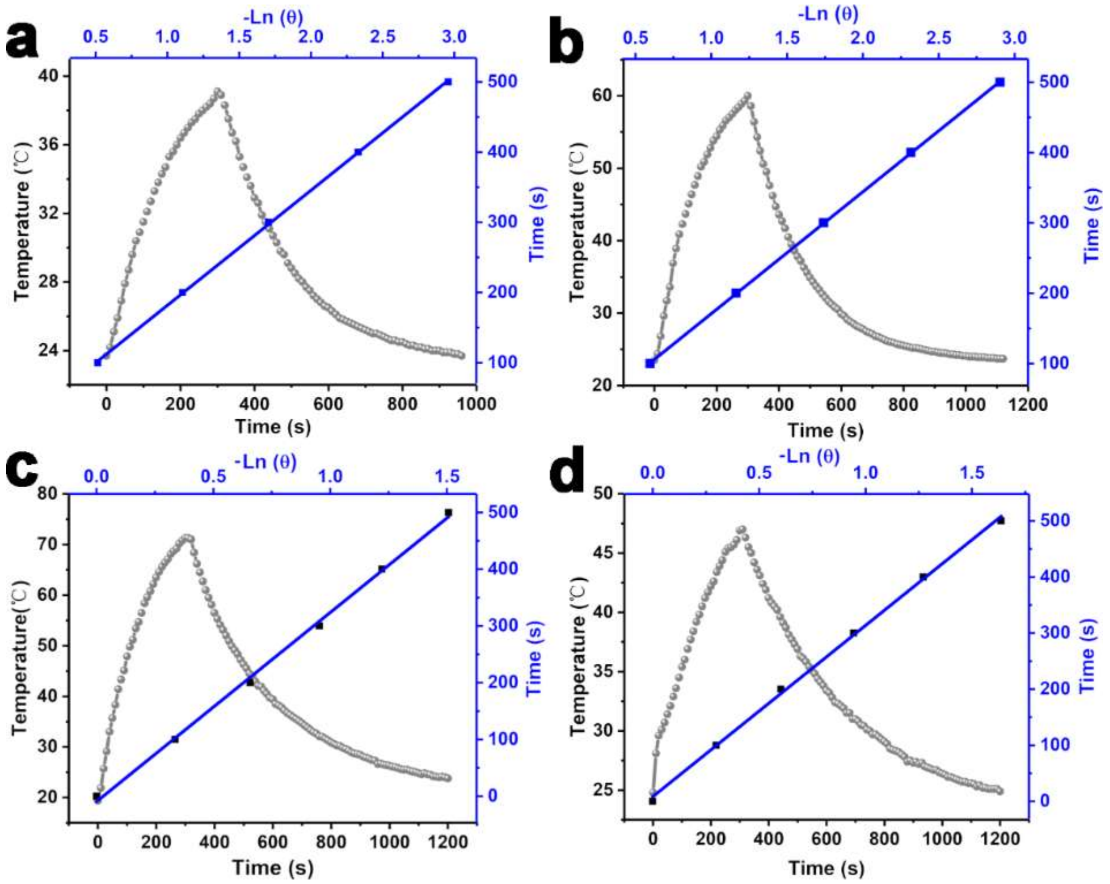

Supplementary Figure 30 Heating-cooling profiles for aqueous dispersions and corresponding photothermal efficiency of **(a)** Fe/CDs@PPSNs-350, **(b)** Fe/CDs@PPSNs-550, **(c)** Fe/CDs@PPSNs and **(d)** CDs@PPSNs. Black line: photothermal effect of an aqueous dispersion under irradiation with 808 nm laser for 5 min, and then the laser was turned off. Blue line: time constant (τ) for the heat transfer from the system determined by applying the linear time data from the cooling period.

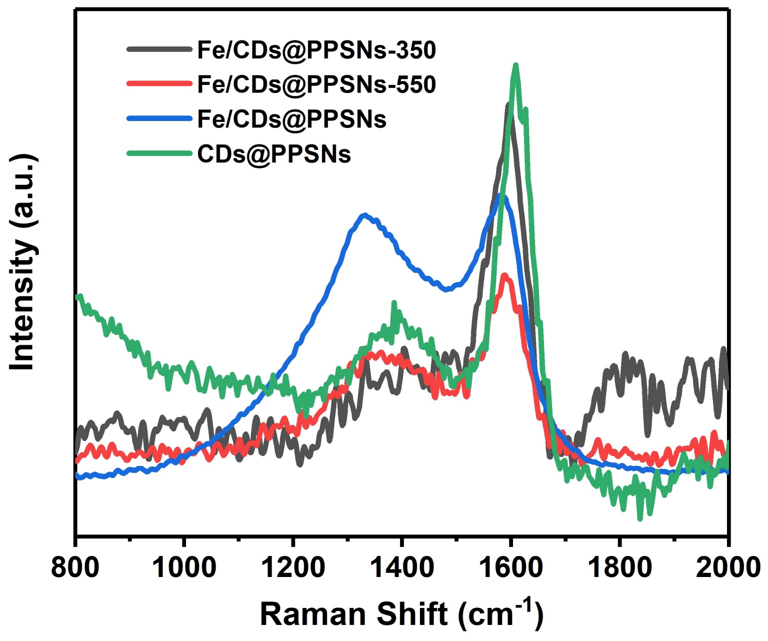

Supplementary Figure 31 Raman spectra of CDs@PPSNs, Fe/CDs@PPSNs-350, Fe/CDs@PPSNs-550 and Fe/CDs@PPSNs.

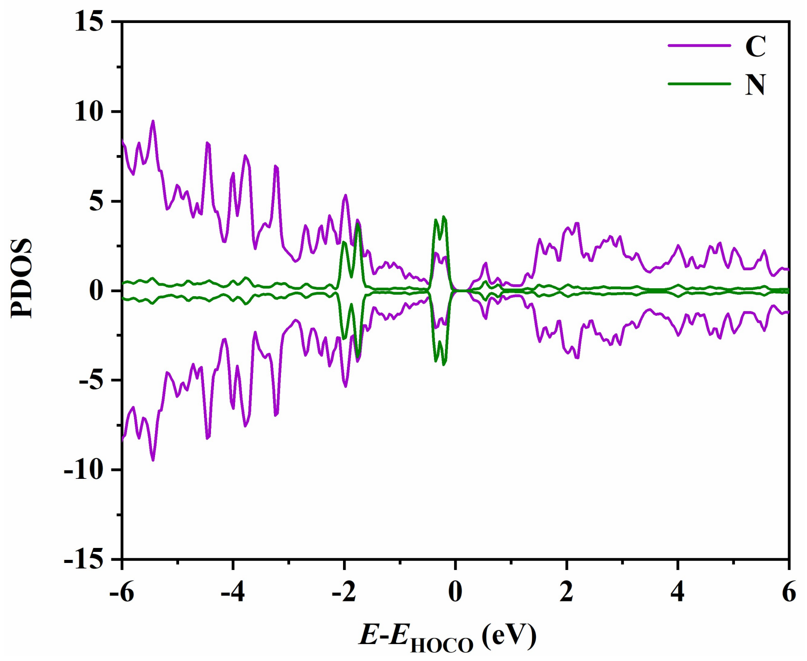

Supplementary Figure 32 The Partial density of states (PDOS) of the defect graphene.

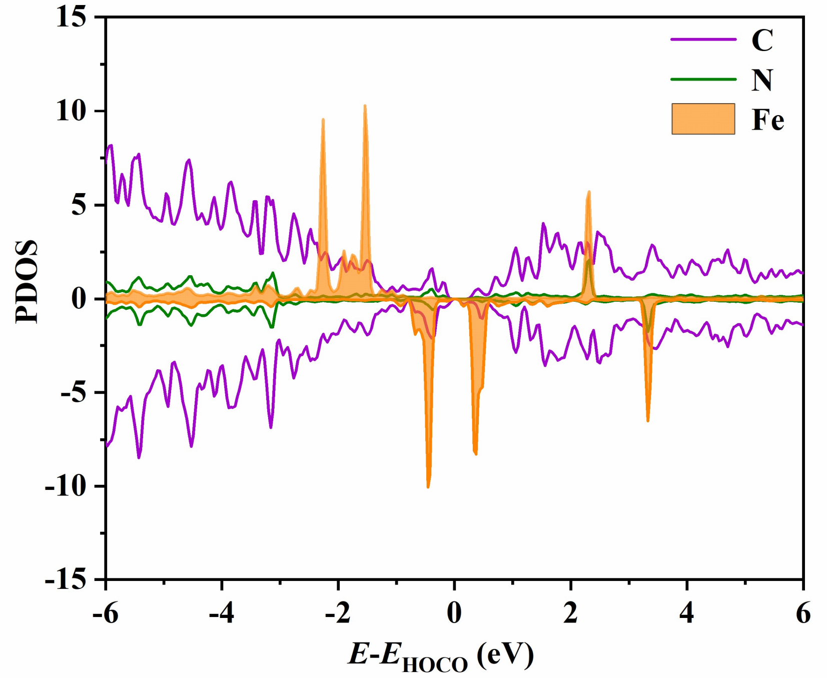

Supplementary Figure 33 The Partial density of states (PDOS) of Fe /CDs@PPSNs.

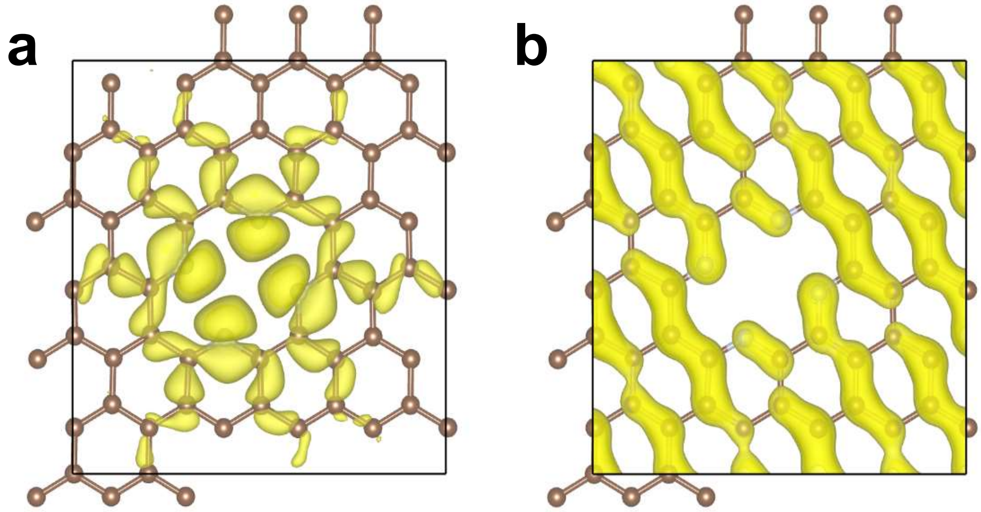

Supplementary Figure 34 Partial charge densities of the **(a)**VBM and **(b)** CBM for the defect graphene.

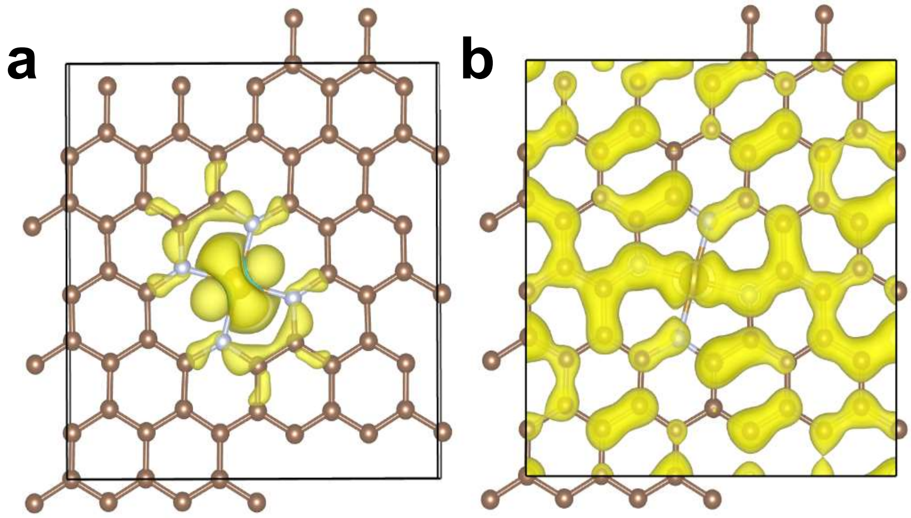

Supplementary Figure 35 Partial charge densities of the **(a)** VBM and **(b)** CBM for Fe/CDs@PPSNs.

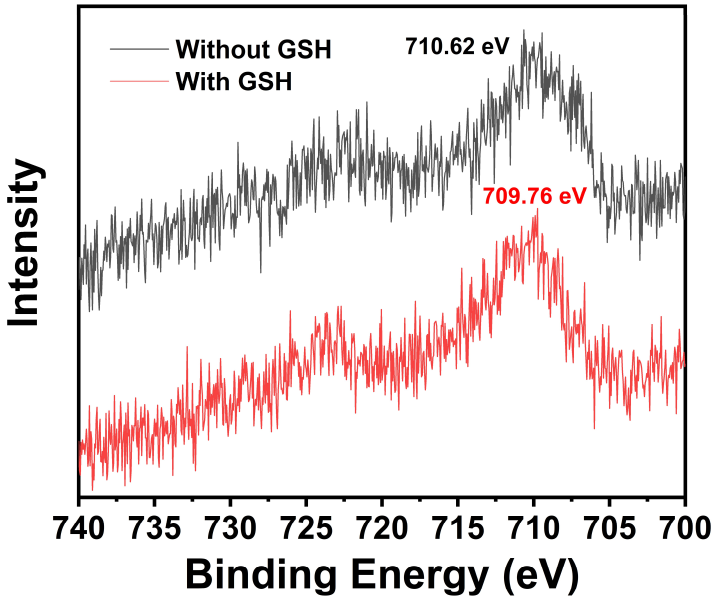

Supplementary Figure 36 The high-resolution Fe *2p* XPS spectra of Fe/CDs@PPSNs treated with/without 10 mM GSH for 1 h.

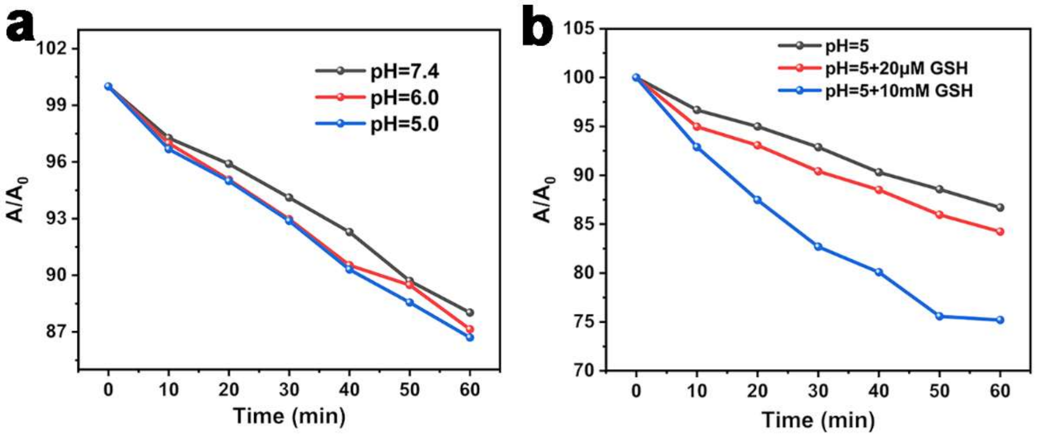

Supplementary Figure 37 Plots of time-dependent degradation curves of MB by IOs/CDs@PPSNs at different **(a)** pH values and **(b)** GSH concentrations.

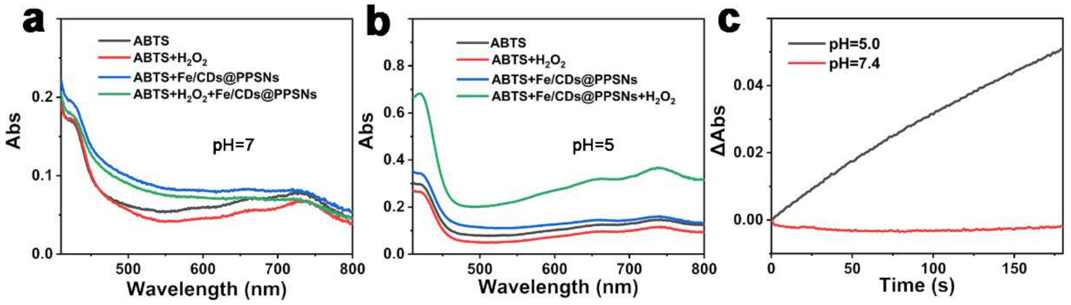

Supplementary Figure 38 The absorbance spectra **(a-b)** and the absorbance at 734 nm **(c)** of different systems by catalyzing the peroxidase substrate 2, 2′-azino-bis (3-ethylbenzthiazoline-6-sulfonic acid) (ABTS) in the presence of H_2O_2 at different pH values.

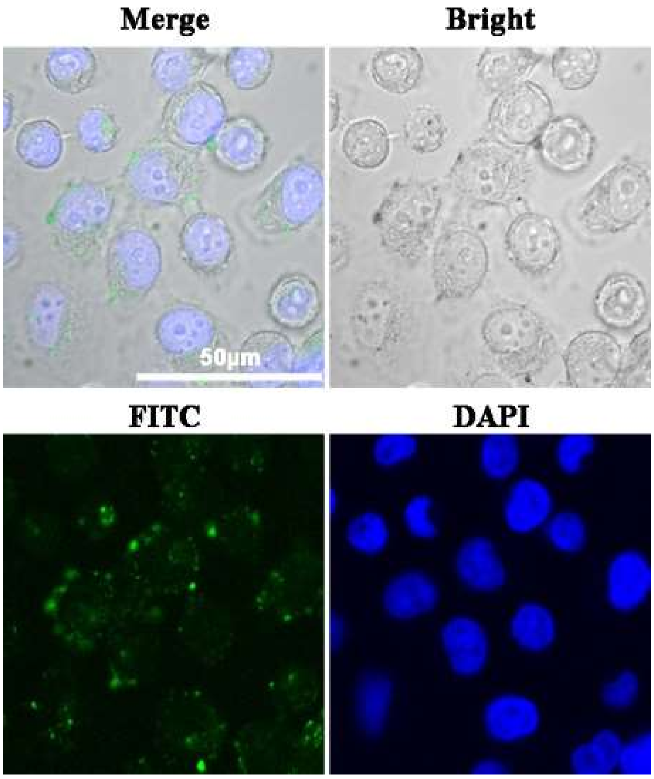

Supplementary Figure 39 The confocal laser scanning microscope (CLSM) images of SMMC-7721 cells after incubation with Fe/CDs@PPSNs for 4 h.

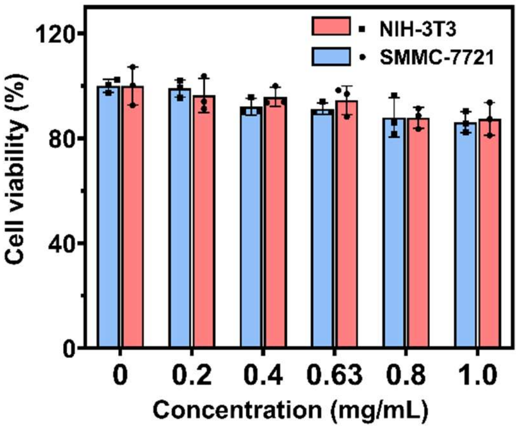

Supplementary Figure 40 The cell viabilities of SMMC-7721 and NIH-3T3 cells treated with Fe/CDs@PPSNs at various particle concentrations. The data are expressed as means ± s. d. from three independent replicates.

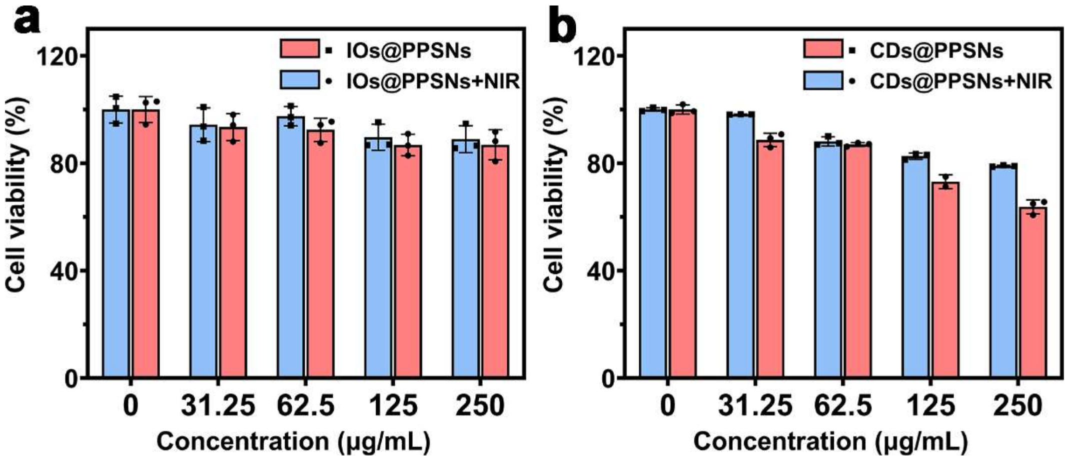

Supplementary Figure 41 SMMC-7721 cell viabilities of **(a)** IOs@PPSNs and **(b)** CDs@PPSNs with or without NIR laser irradiation. The data are expressed as means ± s. d. from three independent replicates.

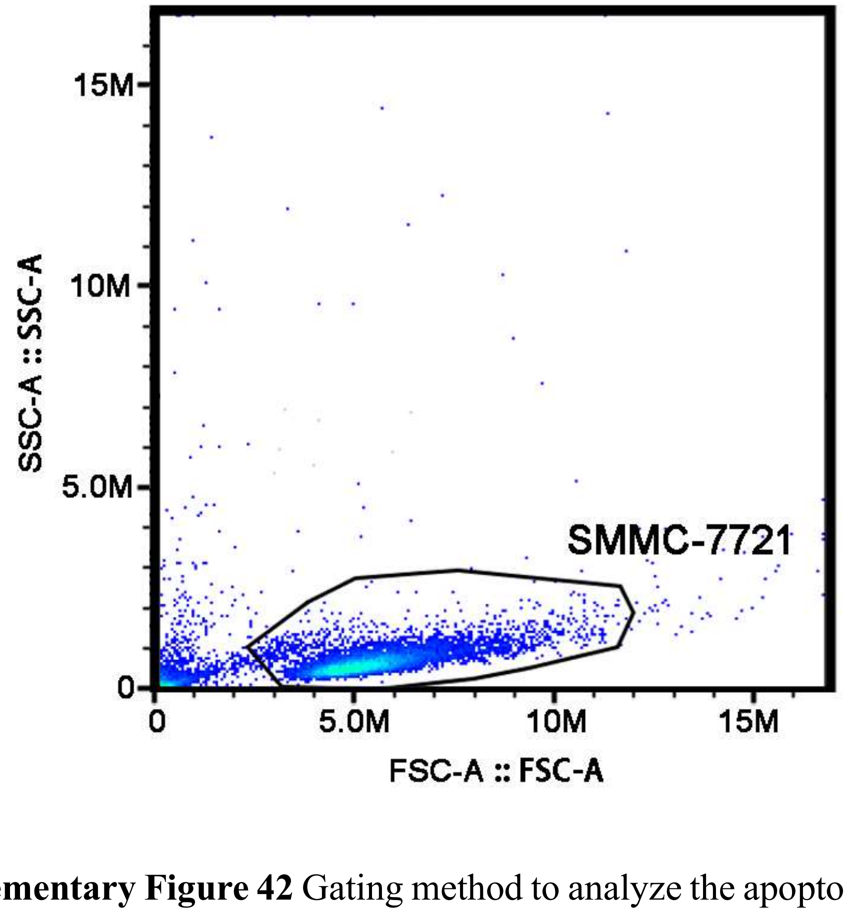

Supplementary Figure 42 Gating method to analyze the apoptosis of cells in Fig. 5f.

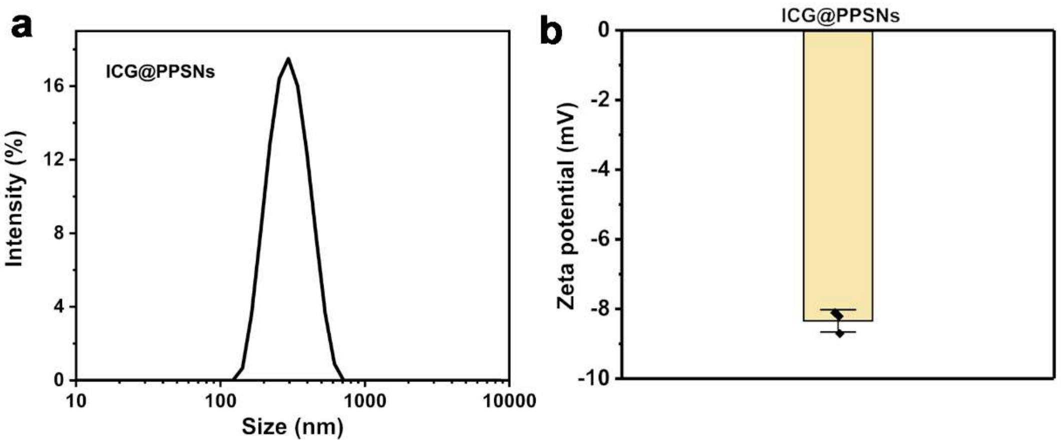

Supplementary Figure 43 (a) The hydrodynamic diameter and **(b)** zeta potential of ICG@PPSNs. The data are expressed as means ± s. d. from three independent replicates.

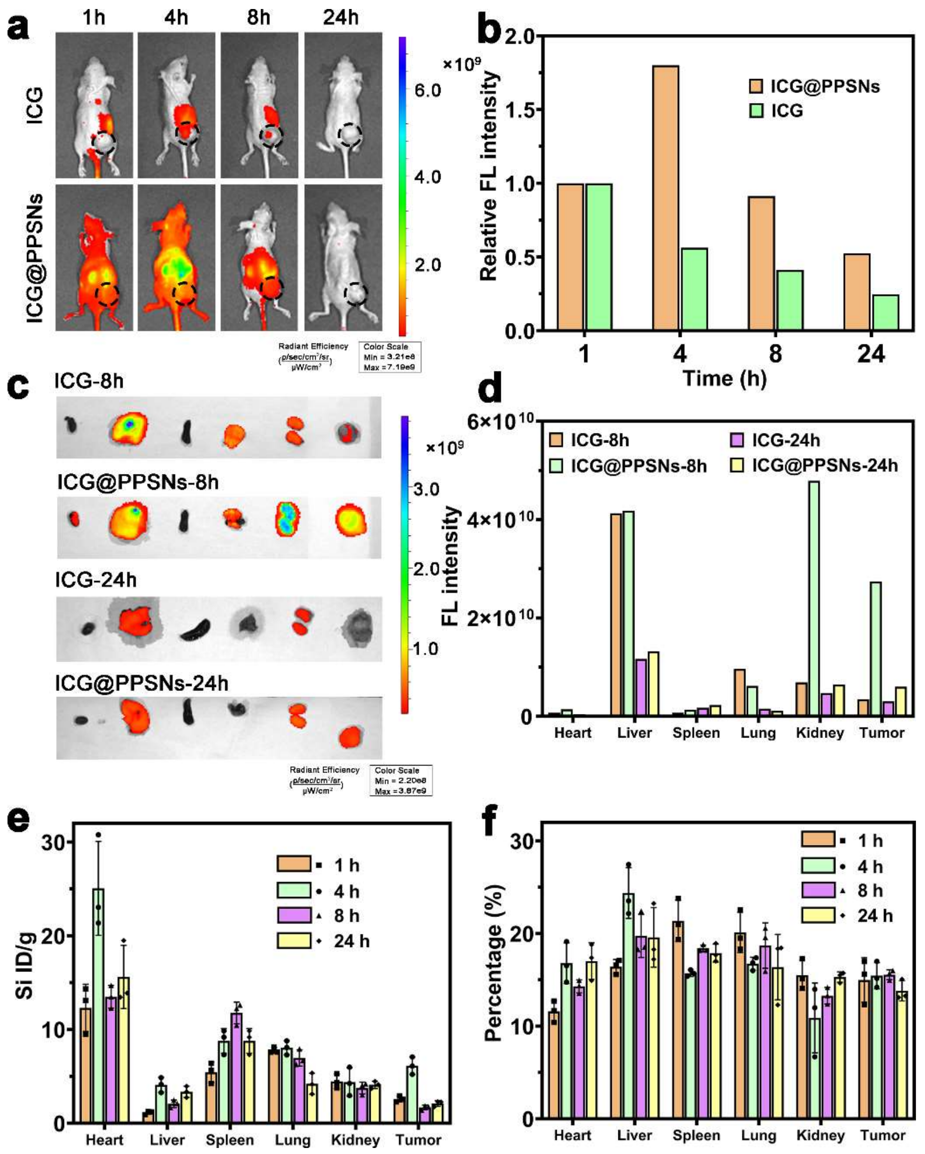

Supplementary Figure 44 (a) In vivo fluorescence images and **(b)** fluorescence intensities of SMMC-7721 tumor-bearing nude mice taken at different time points post i.v. injection of ICG@PPSNs. **(c)** Fluorescence images of major organs after 8 h and 24 h injection of ICG@PPSNs. **(d)** Semiquantitative biodistribution of ICG@PPSNs determined by measuring the ICG fluorescence from ex vivo imaging. **(e)** Biodistribution of Si (% ID of Si per gram of tissues) in main tissues and tumor after intravenous administration of Fe/CDs@PPSNs dispersed in PBS for varied

time intervals (1, 4, 8, and 24 h). **(f)** The total percentage (the amount of each tissue/ total tissues) of Si in main tissues. The data in **e** and **f** are expressed as means ± s. d. from three independent replicates.

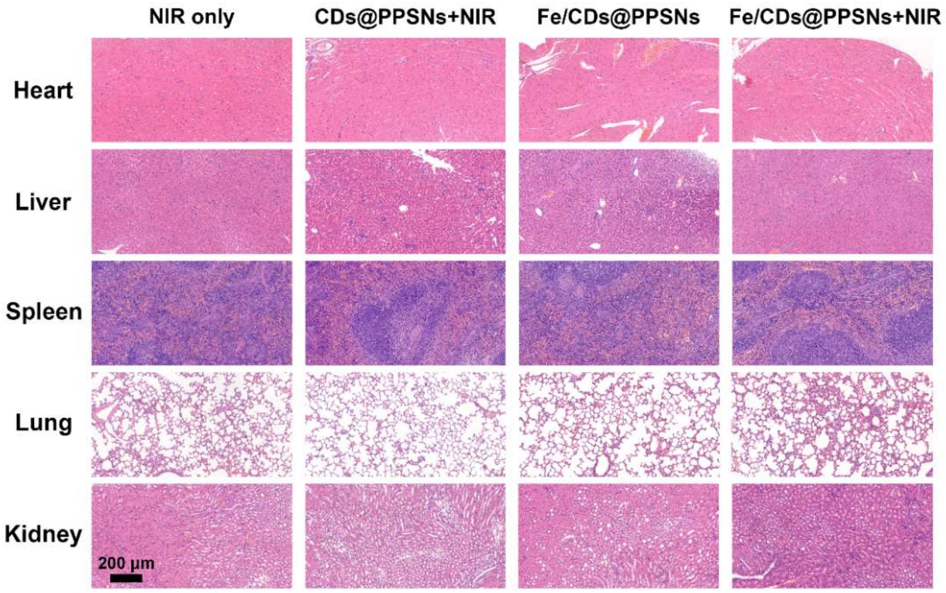

Supplementary Figure 45 H&E staining of major organs (heart, liver, spleen, lung, and kidney) dissected from the nude mice with differently treated groups after 19 days therapeutic period.

Supplementary Table 1 Pore structural parameters and BJH pore size of different samples.

Samples	BET surface (m^2/g)	Pore volume (cm^3/g)	Pore Diameter (nm)
IOs/CDs@PSNs	405	0.70	1.8/13.4
Fe/CDs@PPSNs-350	500	0.86	1.8/14.9
Fe/CDs@PPSNs-550	900	1.24	1.8/14.5
Fe/CDs@PPSNs	518	0.95	1.8/13.7

Supplementary Table 2 Structural parameters extracted from the Fe K-edge EXAFS fitting.

Sample	Shell	N	R	ΔE_0 (eV)	σ^2(10^{-3} Å^2)	R-factor
Fe/CDs@PPSNs	Fe-N	4.4±0.2	1.97±0.01	1.3±0.8	2.9±0.5	0.004

Supplementary Table 3 XPS atomic percentages for the synthesized Fe/CDs@PPSNs.

Sample	C	N	O	Fe
Fe/CDs@PPSNs	5.43	0.75	93.6	0.22

Supplementary Table 4 Photothermal conversion efficiency of various carbon-based PTT agents.

Samples	Power (W/cm^2)	Concentration (ppm)	Time (s)	Temperature increment (°C)	η (%)
DPA-melanin CNSs[1]	2	200	500	33.6	40
Si/C NPs[2]	2	200	490	35	40.7
PGMs[3]	1	80	300	29.6	39.78
MCF[4]	1	50	900	19.1	41.3
PDAC NPs[5]	1	200	600	26.9	22.8
P-MOF[6]	1	50	600	23.6	41
HMCNs[7]	1	200	300	50.3	32
MC-MnO_2[8]	2	25	180	17	27.7
HPFeS_2@C-TA-PEI-GOx-FA[9]	1.5	50	300	17.5	27.2
DOX/HMC-SS-ZnO[10]	2	50	180	54	29.7
**Fe/CDs@PPSNs (This work)**	**1**	**500**	**300**	**28.5**	**58.1**
	**1.5**	**500**	**300**	**41.5**	
	**2**	**500**	**300**	**49.5**	

Supplementary Table 5 Comparison of various photothermal agents and their photothermal conversion efficiencies.

Materials	Photothermal conversion efficiency at 808 nm (%)
Meso-CNs[11]	35.8
Cu_9S_5 nanocrystals[12]	25.7
$Cu_{2-x}Se$ nanocrystals[13]	22
PVP-Bi nanodots[14]	30
FeS@BSA[15]	30.04
Au nanorods[16]	22.1
Gold nanospikes[17]	50.3
UCNPs@mSiO_2-CuS-PEG[18]	30.3
FeCo@C-PEG[19]	26.7
PCB1[20]	42.8

Supplementary Table 6 The structural and photothermal performance parameters of Fe/CDs@PPSNs carbonized at different temperature.

	Fe/CDs@PPSNs-350	Fe/CDs@PPSNs-550	Fe/CDs@PPSNs	CDs@PPSNs
I_D/I_G	0.336	0.654	0.912	0.477
C content (%)	11.455	6.31	6.155	0.97
Mass extinction coefficient (ε, L g^{-1} cm^{-1})	0.25998	0.65432	0.6771	0.18303
ΔT (°C) 2 W/cm^2	17.7	41.1	49.5	25.2

Supplementary Table 7 Comparison of photothermal performance for CDs@PPSNs and Fe/CDs@PPSNs.

	C Concentration (μg/ml)	Sample Concentration (mg/ml)	ΔT(°C)
CDs@PPSNs	15	1.5	35
Fe/CDs@PPSNs	15	0.25	43.6

Supplementary Table 8 TOF values of Fe/CDs@PPSNs incubating with different GSH concentration at pH 5.0.

Time(min)	10	20	30	40	50	60
GSH=20 μM	6.55	4.15	3.16	2.45	2.00	1.80
GSH=10 mM	7.68	5.71	4.36	3.56	3.06	2.70

Supplementary Table 9 TOF values of IOs/CDs@PPSNs incubating with different GSH concentration at pH 5.0.

Time(min)	10	20	30	40	50	60
GSH=20 μM	2.55	1.55	1.43	1.28	1.25	1.17
GSH=10 mM	3.18	2.80	2.58	2.22	2.18	1.85

Supplementary Table 10 The hematological parameters of the mice after treatment with Fe/CDs@PPSNs at the 12th day.

	Control	Fe/CDs@PPSNs
WBC(10^9/L)	5.32±1.30	5.46±0.69
RBC(10^12/L)	9.40±0.34	9.05±0.18
HGB(g/L)	144±5.66	140±2.45
HCT(%)	44.83±1.92	43.83±1.02
MCV(fL)	47.73±1.82	48.47±1.39
MCH(pg)	15.3±0.42	15.47±0.25
MCHC(g/L)	321.33±3.30	319.33±4.50
PLT(10^9/L)	718.33±80.43	1062.00±180.04
RDW-SD(fL)	33.73±2.12	34.57±3.92
RDW-CV(%)	22.37±0.50	22.43±1.32
NEUT#(10^9/L)	1.28±0.34	1.19±0.19
LYMPH#(10^9/L)	3.81±0.97	3.97±0.48
MONO#(10^9/L)	0.04±0.02	0.04±0.02
EO#(10^9/L)	0.19±0.11	0.26±0.13
NEUT%(%)	24.30±4.88	21.80±2.54
LYMPH%(%)	71.73±5.60	72.80±1.64
MONO%(%)	0.70±0.29	0.83±0.41
EO%(%)	3.27±1.28	4.57±1.88

References

1. Liu, Y., et al. Dopamine-Melanin Colloidal Nanospheres: An Efficient Near-Infrared Photothermal Therapeutic Agent for In Vivo Cancer Therapy. *Advanced Materials* **25**, 1353-1359 (2013).
2. Wu, Y., et al. Recent Advances in the Development of Theranostic Nanoparticles for Cardiovascular Diseases. *Nanotheranostics* **5**, 499-514 (2021).
3. Wang, S., et al. Core–Satellite Polydopamine-Gadolinium-Metallofullerene Nanotheranostics for Multimodal Imaging Guided Combination Cancer Therapy. *Advanced Materials* **29**, 1701013 (2017).
4. Wang, D., et al. In Situ One-Pot Synthesis of MOF–Polydopamine Hybrid Nanogels with Enhanced Photothermal Effect for Targeted Cancer Therapy. *Advanced Science* **5**, 1800287 (2018).
5. Xu, K., et al. Polydopamine and ammonium bicarbonate coated and doxorubicin loaded hollow cerium oxide nanoparticles for synergistic tumor therapy. *Nano Research* **12**, 2947-2953 (2019).
6. Wang, L., et al. Exploiting Single Atom Iron Centers in a Porphyrin-like MOF for Efficient Cancer Phototherapy. *ACS Applied Materials & Interfaces* **11**, 35228-35237 (2019).
7. Qiu, Y., et al. Hollow mesoporous carbon nanospheres for imaging-guided light-activated synergistic thermo-chemotherapy. *Nanoscale* **11**, 16351-16361 (2019).
8. Li, X., et al. Mesoporous carbon-manganese nanocomposite for multiple imaging guided oxygen-elevated synergetic therapy. *Journal of Controlled Release* **319**, 104-118 (2020).
9. Wu, F., et al. Hollow Porous Carbon Coated FeS2-Based Nanocatalysts for Multimodal Imaging-Guided Photothermal, Starvation, and Triple-Enhanced Chemodynamic Therapy of Cancer. *ACS Applied Materials & Interfaces* **12**, 10142-10155 (2020).
10. Feng, S., et al. Triple stimuli-responsive ZnO quantum dots-conjugated hollow mesoporous carbon nanoplatform for NIR-induced dual model antitumor therapy. *Journal of Colloid and Interface Science* **559**, 51-64 (2020).
11. Zhou, L., et al. Mesoporous Carbon Nanospheres as a Multifunctional Carrier for Cancer Theranostics. *Theranostics* **8**, 663-675 (2018).
12. Fu, C., et al. Microwave-Activated Mn-Doped Zirconium Metal–Organic Framework Nanocubes for Highly Effective Combination of Microwave Dynamic and

Thermal Therapies Against Cancer. *ACS Nano* **12**, 2201-2210 (2018).
13. Zhang, Z., Wang, J. and Chen, C. Near-Infrared Light-Mediated Nanoplatforms for Cancer Thermo-Chemotherapy and Optical Imaging. *Advanced Materials* **25**, 3869-3880 (2013).
14. Lei, P., et al. Ultrafast Synthesis of Ultrasmall Poly(Vinylpyrrolidone)-Protected Bismuth Nanodots as a Multifunctional Theranostic Agent for In Vivo Dual-Modal CT/Photothermal-Imaging-Guided Photothermal Therapy. *Advanced Functional Materials* **27**, 1702018 (2017).
15. Yang, W., et al. Albumin-constrained large-scale synthesis of renal clearable ferrous sulfide quantum dots for T1-Weighted MR imaging and phototheranostics of tumors. *Biomaterials* **255**, 120186 (2020).
16. Zeng, J., Goldfeld, D. and Xia, Y. A plasmon-assisted optofluidic (PAOF) system for measuring the photothermal conversion efficiencies of gold nanostructures and controlling an electrical switch. *Angewandte Chemie International Edition* **52**, 4169-73 (2013).
17. Ma, N., et al. Enhanced Radiosensitization of Gold Nanospikes via Hyperthermia in Combined Cancer Radiation and Photothermal Therapy. *ACS Applied Materials & Interfaces* **8**, 28480-28494 (2016).
18. Xu, M., et al. An intelligent nanoplatform for imaging-guided photodynamic/photothermal/chemo-therapy based on upconversion nanoparticles and CuS integrated black phosphorus. *Chemical Engineering Journal* **382**, 122822 (2020).
19. Song, G., et al. Carbon-coated FeCo nanoparticles as sensitive magnetic-particle-imaging tracers with photothermal and magnetothermal properties. *Nature Biomedical Engineering* **4**, 325-334 (2020).
20. Li, J., et al. Semiconducting Polymer Nanoenzymes with Photothermic Activity for Enhanced Cancer Therapy. *Angewandte Chemie International Edition* **57**, 3995-3998 (2018).
